# Extended polyene formation by a cryptic iterative polyketide synthase from *Rhodococcus*†

**DOI:** 10.1039/d4cc04963b

**Published:** 2024-10-29

**Authors:** Panward Prasongpholchai, Sam Tucker, Charles Burgess, Robert Jenkins, Ina Wilkening, Christophe Corre, Lijiang Song, Manuela Tosin

**Affiliations:** a Department of Chemistry, University of Warwick Gibbet Hill Road Coventry CV4 7AL UK pan.prasongpholchai@warwick.ac.uk m.tosin@warwick.ac.uk; b School of Life Sciences, University of Warwick Gibbet Hill Road Coventry CV4 7AL UK

## Abstract

Many reactive intermediates leading to high value molecules are biosynthesised by multifunctional enzymes in Actinobacteria. Herein we report the workings of a cryptic iterative polyketide synthase (iPKS) from the marine microorganism *Rhodococcus erythropolis* PR4. The iPKS generates extended polyenes up to C22 nonaenes, preluding novel chemistry and biology.

## Abstract 2

Investigations of the workings of a newly identified iterative polyketide synthase from *Rhodococcus erythropolis* PR4 reveal its ability to synthesise extended polyenes.

## Graphical abstract

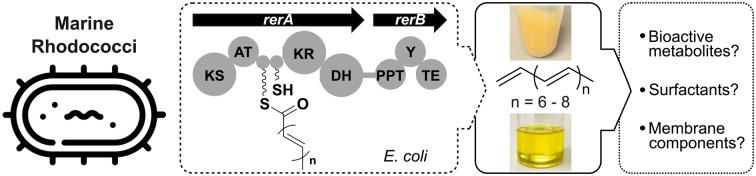

Polyenes are structurally intriguing molecules featuring multiple linear conjugated double bonds. In nature, they are widely utilised as moieties for photoprotection, and they exert antioxidant, antibiotic and antifungal properties, either on their own or as part of complex natural products.^1–4^ Synthetically, polyenes constitute highly reactive intermediates for the generation of bioactive linear and cyclised scaffolds, as well as conductive polymers.^5^ In recent years, they have also gathered much interest as precursors of aviation biofuels.^6^ Yet, polyenes remain to date amongst the most challenging moieties to access for chemical synthesis and utilisation due to their inherent instability. In nature, polyenes can be biosynthesised by different classes of enzymes such as terpene synthases and polyketide synthases (PKSs). The latter can operate in a modular or iterative fashion, utilising activated malonyl and acyl building blocks, and decarboxylative Claisen condensation to build polyene frameworks in bioactive molecules such as the antifungal nystatin and amphotericin B, and aryl polyene pigments. Genes encoding for polyketide biosynthesis are predominantly found in Actinomycetes, with genome analysis of newly discovered or underexploited microorganisms progressively unveiling novel biosynthetic potential leading to valuable products and chemistry.^7^*Rhodococcus erythropolis* PR4 (NBRC 100887) is a Gram-positive, high G + C content marine bacterium first isolated from the Pacific Ocean and known for its ability to degrade xenobiotics.^8^ Bioinformatics analyses of its genome *via* antiSMASH^9^ reveal the presence of 19 biosynthetic gene clusters (BGCs, Table S1, ESI†). Amongst these, only two have been characterised and found to be responsible for the production of peptides such as the antibacterial corynecins (structurally related to chloramphenicol)^10^ and the siderophore heterobactins.^11^ During the course of our investigations, BGC #3 (Table S1 and Fig. S1, ESI†) of 46 kb in size and encoding for an iterative polyketide synthase (iPKS) attracted our attention due to the presence of (i) two tandem acyl carrier protein (ACP) domains- a common feature of bacterial *de novo* polyunsaturated fatty acid (PUFA) synthases^12^ leading to fatty acid products such as docosahexa enoic acid (1, Fig. 1A); and (ii) a ketosynthase (KS) domain showing high similarity to iPKSs responsible the production of extremely rare and potent enediyne antitumor antibiotics, exemplified by dynemicin A (2, Fig. 1A).^1^ These intriguing features prompted us to further investigate BGC#3 and the workings of its core components. Amongst the 39 putative genes in the cluster, RER_RS03860 (herein newly named as *rerA*) encodes for a type I iterative PKS, RerA, comprising a KS domain, an acyl transferase (AT) domain, two tandem ACP domains (ACP1 and ACP2), a ketoreductase (KR) domain and a dehydratase (DH) domain (Fig. 1B). Downstream to *rerA* is RER_RS03865 (hereinafter referred to as *rerB*), which encodes for a multifunctional protein, RerB, featuring a phosphopantetheinyl transferase (PPTase) domain, an unknown domain Y and a thioesterase (TE) domain. To elucidate the nature of the product(s) associated with RerA and RerB, we cloned *rerA* and *rerB* from *R. erythropolis* PR4 genomic DNA, inserting the genes into pET28-based plasmids and heterologously overexpressing each construct in *E. coli* BL21(DE3) cells. In parallel, we also coexpressed *rerA* and *rerB* (cloned and inserted in a pETDuet vector) as we envisaged that the PPTase domain of RerB may be essential for the post-translational modification of *apo*-ACP domains into functional *holo* carrier proteins. When *E. coli* BL21(DE3) cells harbouring pETDuet-*rerA-rerB* were cultured at 20 °C overnight post-induction, we noticed an unusual pale-yellow colouration of the cell pellet, indicating the possible presence of chromophore products (Fig. 2A). This colouration was absent from the cell pellet of cultures grown at 30 °C and of cultures overexpressing either *rerA* or *rerB* alone. As both *rerA* and *rerB* were coexpressed using a bicistronic vector (pETDuet), the level of two recombinant proteins produced might not be the same, and this may affect the titre and the nature of the resulting product. We, therefore, aimed to address the issue by linking *rerA* and *rerB via* a flexible 5 amino acids linker (Gly–Ser–Gly–Ser–Gly) to yield one single recombinant RerA-linker-RerB megaenzyme, hereinafter denoted as RerAB. When pET-*rerAB* was overexpressed in *E. coli* in the same conditions, we noticed a more intense yellow colouration for the cell pellet compared to that previously obtained from pETDuet-*rerA-rerB* cultures (Fig. 2A). To confirm the involvement of iPKS catalysis in yellow colour formation, site-directed mutagenesis was performed to inactivate selected amino acid residues within RerA/B domains (Fig. S2, ESI†). Inactivation of the KS and PPTase domains resulted in colourless cell pellets (Fig. S4, ESI†), whereas TE domain inactivation led to orange coloured cell pellets, possibly related to enzyme- bound species (Fig. 2A).

**Fig. 1:**
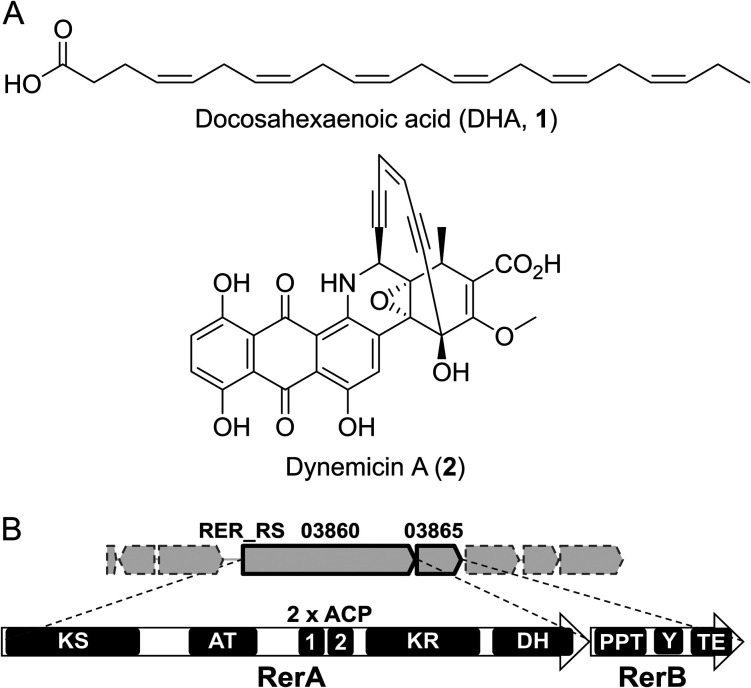
(A) Chemical structures of iPKS products docosahexaenoic acid (1) and dynemicin A (2). (B) Domain organisation of newly identified iPKS RerA and PKS-related protein RerB from *R. erythropolis* PR4 genome (this work). RerA-B share similarities with iPKSs responsible for the making 1 and 2 (Fig. S2 and S3, ESI†).

**Fig. 2:**
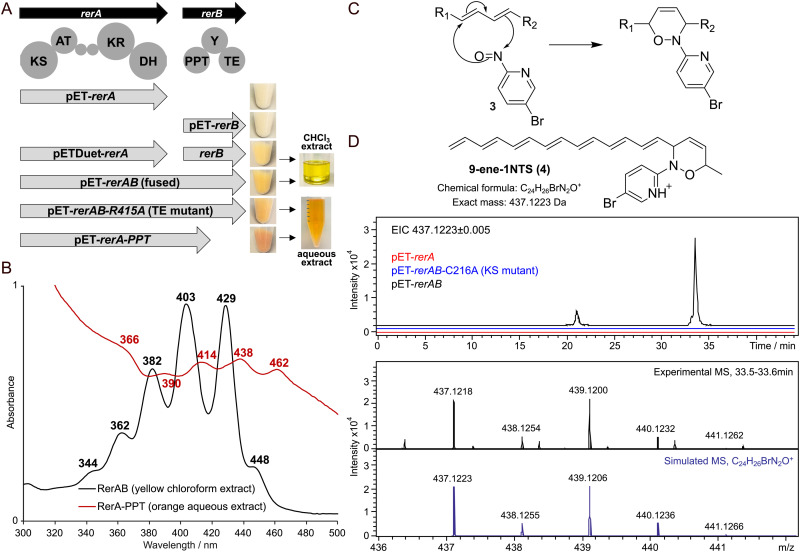
(A) Constructs generated for the heterologous expression of *rerA-B* in *E. coli* with corresponding cell pellets and extracts obtained. (B) UV-vis profiles of chloroform extracts from lyophilised *E. coli* cells expressing pET-*rerAB* (black trace), and of aqueous extracts from *E. coli* cells expressing pET-*rerA-PPT* (red trace). (C) Mechanism of Diels–Alder reaction between the nitroso group of 3^13^ and a diene moiety. (D) Putative structure of a derivatised nonaene adduct (9-ene-1NTS, 4) detected by high resolution LC-MS analysis upon derivatisation with 3 and its extracted ion chromatogram (EIC). The two peaks detected at 21 and 33 min respectively may be isomers.

We then aimed to extract and characterise the yellow-orange pigmented compounds. Several methods were attempted, including organic extraction from whole cell cultures, supernatants, wet cell pellets and lyophilised cell pellets. The only effective procedure to isolate coloured small molecules was the use of chloroform to extract lyophilised *E. coli* cell pellets. The UV-Vis spectrum of chloroform yellow extracts exhibited multiple peak absorption bands at *λ*_max_ of 362, 382, 403 and 429 nm (Fig. 2B, black trace), indicating the presence of a highly conjugated system of polyenic nature. Further direct chemical characterisation of the extracts by LC-MS and NMR proved inconclusive, likely due to the low amount of material effectively isolated and its possible instability to temperature and light. We therefore envisaged that nitroso (NTS) pyridine probes such as 3 (Fig. 2C) could be utilised for Diels–Alder reactions to convert conjugated alkenes into more stable adducts, enabling their labelling and characterisation *via* LC-MS. The bromo-nitroso pyridine probe 3 was synthesised as previously reported^13^ and utilised for the characterisation of the yellow chloroform crude extracts. The LC-MS results of this derivatisation showed the presence of polyenes ranging from 6 to 9 conjugated double bonds, including the unprecedented nonadeca-1,3,5,7,9,11,13,15,17-nonaene and heptadeca-1,3,5,7,9,11,13,15-octaene, and of related polyenones. Proposed nitroso adducts for all these species (*e.g.*4) are shown in Fig. 2D and in the ESI† (Fig. S5–S10 and Table S3). In parallel to extracted small molecule characterisation, attempts were also made to elucidate the enzyme-bound, orange-coloured species, generated by the overexpression of pET-*rerA-PPT* in *E. coli*. The UV-Vis profile of orange aqueous samples (Fig. 2B, red trace) showed a similar polyene-like absorption pattern, red-shifted by 22–35 nm in comparison to that of yellow small molecule extracts. Protein coexpression experiments *in E. coli* were devised to attempt the capture of enzyme-bound intermediates onto discrete ACP domain *in trans* (Fig. 3A). Two compatible plasmids were cotransformed into *E. coli* BL21(DE3) cells: one encoding for ACP-inactivated RerA-PPT in an untagged form (pACYC-*rerA*-PPT-ACPsMut, 235 kDa); and the other encoding for a discrete active hexahistidine-tagged ACP domain (pET-*ReACP1*, 11.5 kDa or pET-R*eACP2*, 12.9 kDa; Fig. S20, ESI†). Upon protein coexpression and Ni^2+^ affinity purification of hexahistidine (His_6_)-tagged proteins, ReACP1 and ReACP2 were isolated, both featuring bright orange colourations. Gratifyingly, intact protein ESI-MS analysis of these individual proteins revealed the presence of variable long-chain polyenoyl species bound to the ACP domain (Fig. 3B and Fig. S11, ESI†). All these species (up to C22 in chain length) are unprecedented and consistent with hepta- to nona- polyene formation as detected from derivatised extracts (Fig. 2). The nature of the ACP-bound species (Fig. 3B and Fig. S11, ESI†) is also consistent with free polyene and β-ketopolyene generation resulting from TE domain-catalysed hydrolysis and subsequent decarboxylation and dehydration of β-hydroxy/keto acyl species as reported for enediyne formation (Fig. S12, ESI†).^14,15^ To complement our findings, we reconstituted the activity of recombinant RerA and RerB enzymes *in vitro*. RerA (219 kDa) and RerB (61.5 kDa) were successfully expressed and purified from *E. coli* in soluble forms (Fig. S13 and S21, ESI†), and upon their incubation with malonyl-CoA, Mg^2+^ and NADPH in a buffer (20 mM Tris, 100 mM NaCl, pH 7.5) at room temperature, a bright yellow colour developed in solution within 3 minutes (Fig. S14, ESI†). By monitoring assay product formation at 414 and 438 nm, we found that absorbance at these wavelengths peaked at 25–35 min and steadily declined thereafter (Fig. S15, ESI†), indicating that the formed polyenes are not stable for longer periods of time. Lastly, to gather stepwise mechanistic details for early-stage polyene formation, we utilised non-hydrolysable chain termination probes developed by our group to intercept and off-load polyketide intermediates from the working recombinant iPKS.^16,17^ Two 4,5-dimethoxy-2-nitrobenzyl (DMNB)-protected probes, 5 and 6, were newly synthesised from γ-amino butyric acid (Scheme S1 and Fig. S16, 17, ESI†) and uncaged *via* photolysis at 365 nm prior to their addition to *in vitro* assays with RerA and RerB (Fig. 3C). LC-MS analyses of organic extracts revealed interception of polyketide intermediates by the uncaged probes 7–8, with polyenones being the most abundant intercepted species (characterised by HR-MS^2^ as previously reported by us;^16,17^ species summary and examples given in Fig. 3C and Fig. S18, S19, ESI†). 7 proved the most suitable ACP-malonate mimic in capturing intermediate species from RerAB, whereas the fluorinated probe 8 was only able to intercept intermediates related to diketide formation and processing. This may indicate that the iPKS is not capable of utilising substituted malonyl derivatives for further carbon chain extension. Also, the longest polyketides that could be intercepted in these experiments were hexa- and penta-ene species. It is possible that the length of the *N*-decanoyl moiety may limit the ability of probes such as 7–8 to react with all enzyme-bound species, especially when the latter already feature extended chain lengths (Fig. S11, ESI†) and may occupy most of the KS active site. The identification and characterisation of *Rhodococcus* genes associated to type I iterative polyketide biosynthesis, together with the proven ability of their related proteins to generate novel polyenes *in vivo* and *in vitro*, is unprecedented and highly significant. Rhodococci are known to harbour a plethora of biocatalysts and molecules, many of which have found applications but are still of unknown origins.^18^ At this point in time, the complete and true nature of the rerAB cluster product(s) in its native host remain under investigation in our lab. Nonetheless, we have herein revealed the stepwise workings of its core proteins, RerA and RerB, which can biosynthesise extended conjugated frameworks preluding novel chemistry and biology.

**Fig. 3:**
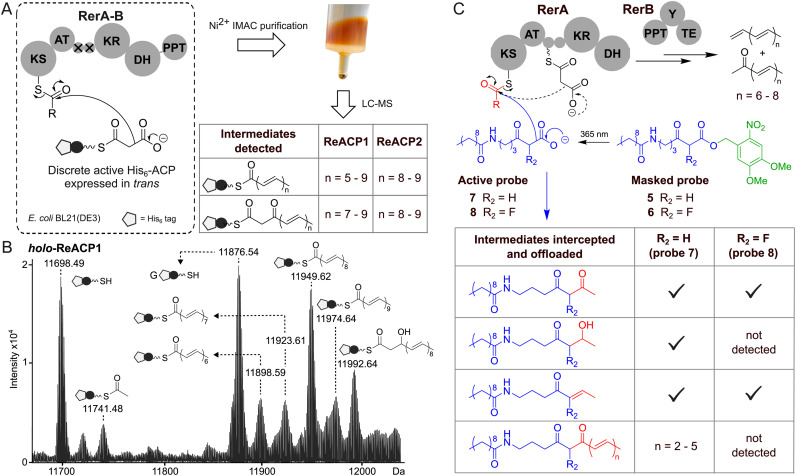
(A) Illustration of coexpression of RerAB bearing inactive ACP domains and a discrete active ACP domain (ACP1 or ACP2) *in trans* in *E. coli* BL21(DE3), leading to the accumulation of orange-coloured ACP-bound species that can be isolated from the *E. coli* cultures by Ni^2+^ IMAC affinity chromatography. (B) Deconvoluted mass spectrum of intact recombinant His_6_-ReACP1 purified from protein coexpression experiments showed different chain length polyketide/polyene intermediates bound to the ACP domain. G denotes the gluconoylated version of the recombinant protein (further details in the ESI†). (C) Interception and offloading of iPKS-bound polyketide intermediates from RerA and RerB from *in vitro* assays using chemical chain termination probes 7 and 8 (generated by photolysis of 5 and 6 at 365 nm).

We gratefully acknowledge the University of Warwick Chancellor's International Scholarship (awarded to P. P.); the Warwick Monash Alliance (PhD studentship to S. T.); the Midlands Integrative Biosciences Training Partnership (PhD studentship to C. B. and Research Experience Placement bursary to S. T.); EPSRC (DTA PhD studentship to R. J.); the European Commission (FP7 Marie Curie Intra-European Fellowship to I. W., FP7-PEOPLE-2013-IEF, project n. 628069); Dr Matthew Jenner (Chemistry Department, Warwick) for early assistance with intact protein MS data acquisition; and Dr Cleidi Zampronio (School of Life Sciences, Warwick) for assistance with LC-HRMS^*n*^ Orbitrap Fusion analyses.

## Data availability

The data supporting this article have been included as part of the ESI.†

## Conflicts of interest

There are no conflicts to declare.

## Supplementary Material

CC-060-D4CC04963B-s001
