# Supplementary material for: Extended polyene formation by a cryptic iterative polyketide synthase from Rhodococcus
Source: Chem Commun (Camb). 2024 Oct 29;60(95):14085–8. doi: 10.1039/d4cc04963b (PMC11563203; doi:10.1039/d4cc04963b)
Supplement: CC-060-D4CC04963B-s001 [file CC-060-D4CC04963B-s001.pdf]

Supplementary Information (SI) for Chemical Communications.
This journal is © The Royal Society of Chemistry 2024

Supporting Information

Extended polyene formation by a cryptic iterative polyketide synthase from *Rhodococcus*

Panward Prasongpholchai,* Sam Tucker, Charles Burgess, Robert Jenkins, Ina Wilkening, Christophe Corre, Lijiang Song, and Manuela Tosin*

Table of contents

Supplementary Figures 3
Supplementary Tables 21
Chemistry general methods and materials 24
Compound analysis and characterisation 24
Synthesis of the nitrosopyridine (NTS) probe 3 24
Synthesis and characterisation of photolabile chemical probes 24
Synthesis of probe 5 25
Synthesis of probe 6 25
General methods and materials for molecular biology 26
List of plasmid vectors used 26
Bioinformatic tools 26
Accession number 26
List of plasmids generated 27
List of primers used in this study 29
General cloning procedure 30
Site-directed mutagenesis 30
Recombinant protein overproduction in *E. coli* 30
Protein purification 30
Extraction of yellow-coloured products 31
Extraction of orange protein solution from RerA-PPT and RerA-PPT-Y 31
Characterisation of coloured material by UV-Vis spectroscopy 31
In vitro reconstitution of core iPKS enzymes (RerA-B) 31
Monitoring of *in vitro* iPKS activity by UV-Vis spectroscopy 31
Use of photolabile probes in assays with recombinant iPKS enzymes 32
Purification and characterisation of orange enzyme-bound species from protein coexpression in *E. coli* .. 32
LC-MS^n analyses 34
Analyses of recombinant proteins and coloured ACP domains 34
Analyses of small molecules 34
Orbitrap Fusion analyses of small molecules 34
Protein sequences of *R. erythropolis* PR4-related constructs generated for this work 35
p28T-rerA (KS-AT-ACP1-ACP2-KR-DH) 35
pET28a-rerB (PPTase-Y-TE) 36
p28T-rerAB (fused) 36
p28T-RePPT (standalone PPTase domain from RerB) 37
p28T-ReACP1 (standalone ACP1 domain from RerA) 37
p28T-ReACP2 (standalone ACP2 domain from RerA) 37
References 38

Supplementary Figures

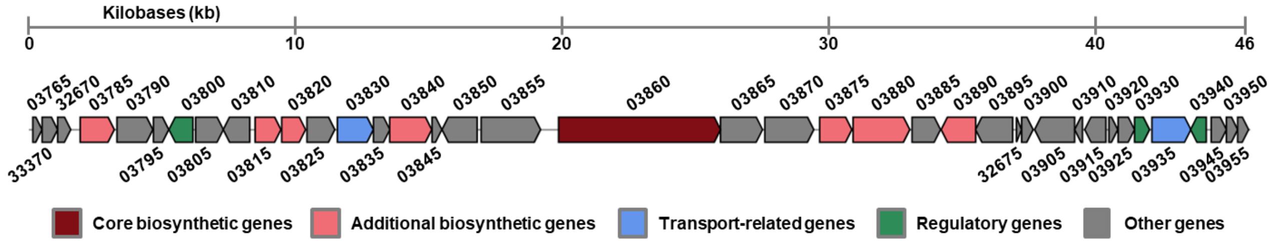

Figure S1 Genetic organisation of genes from *Rhodococcus erythropolis* PR4 BGC no. 3 spanning over 46 kb in size and comprising 39 putative genes. The putative function of each gene is shown in Table S2.

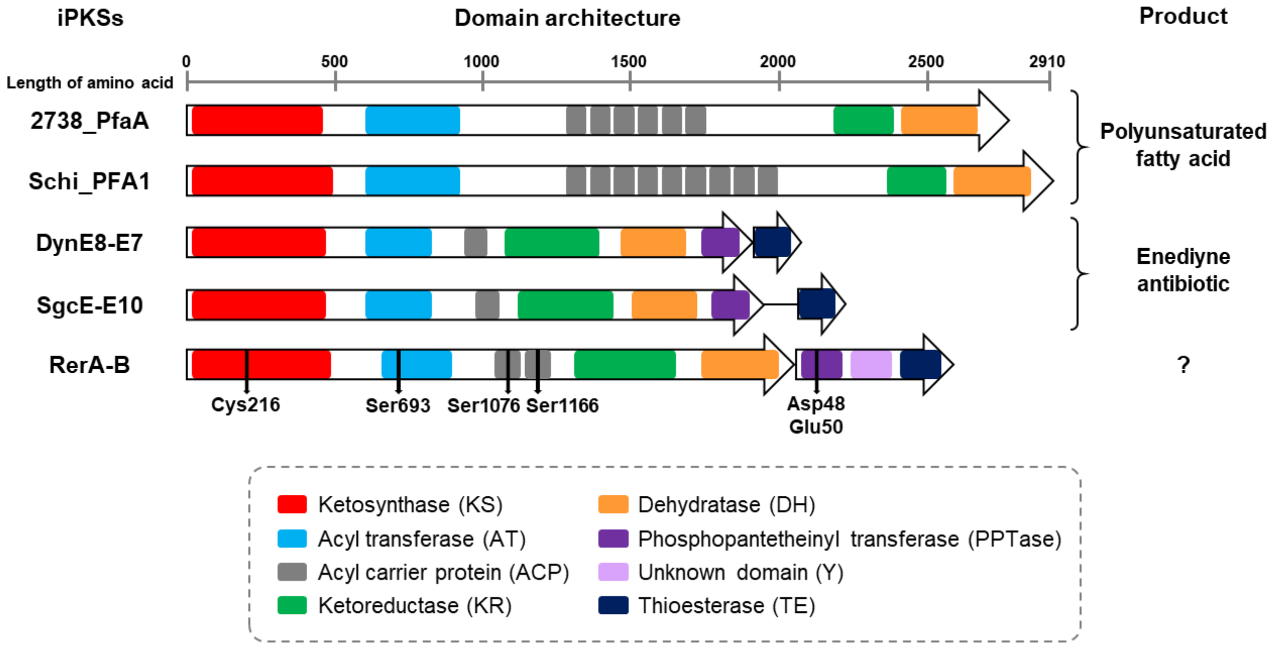

Figure S2 Domain organisation of selected iPKSs: PUFA synthases (2738_PfaA: PfaA from *Shewanella* sp. SCRC 2738; Schi_PFA1: PFA1 from *Schizochytrium* sp. ATCC 20888), enediyne core synthases (DynE8-E7 from *Micromonospora chersina*; SgcE-E10 from *Streptomyces globisporus*); and RerA-B from *Rhodococcus erythropolis* PR4. The position of critical catalytic residues is also shown for RerA and RerB.

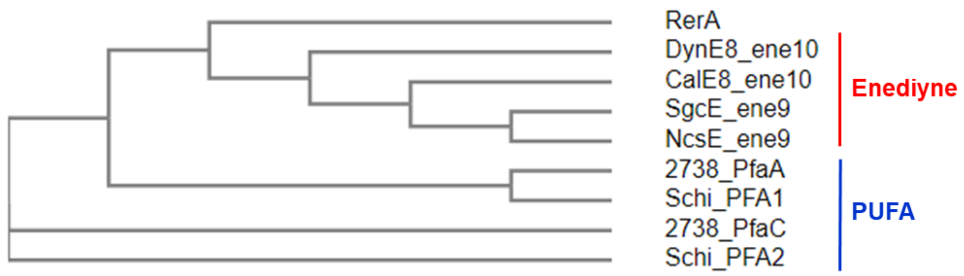

Figure S3 Phylogenetic tree of selected KS domains from characterised enediyne core synthase and PUFA synthase, generated using Clustal Omega. ene10: KS domain of iPKS from 10-membered enediyne core synthase; ene9: KS domain of iPKS from 9-membered enediyne core synthase; Pfa/PFA: KS domain of PUFA synthase.

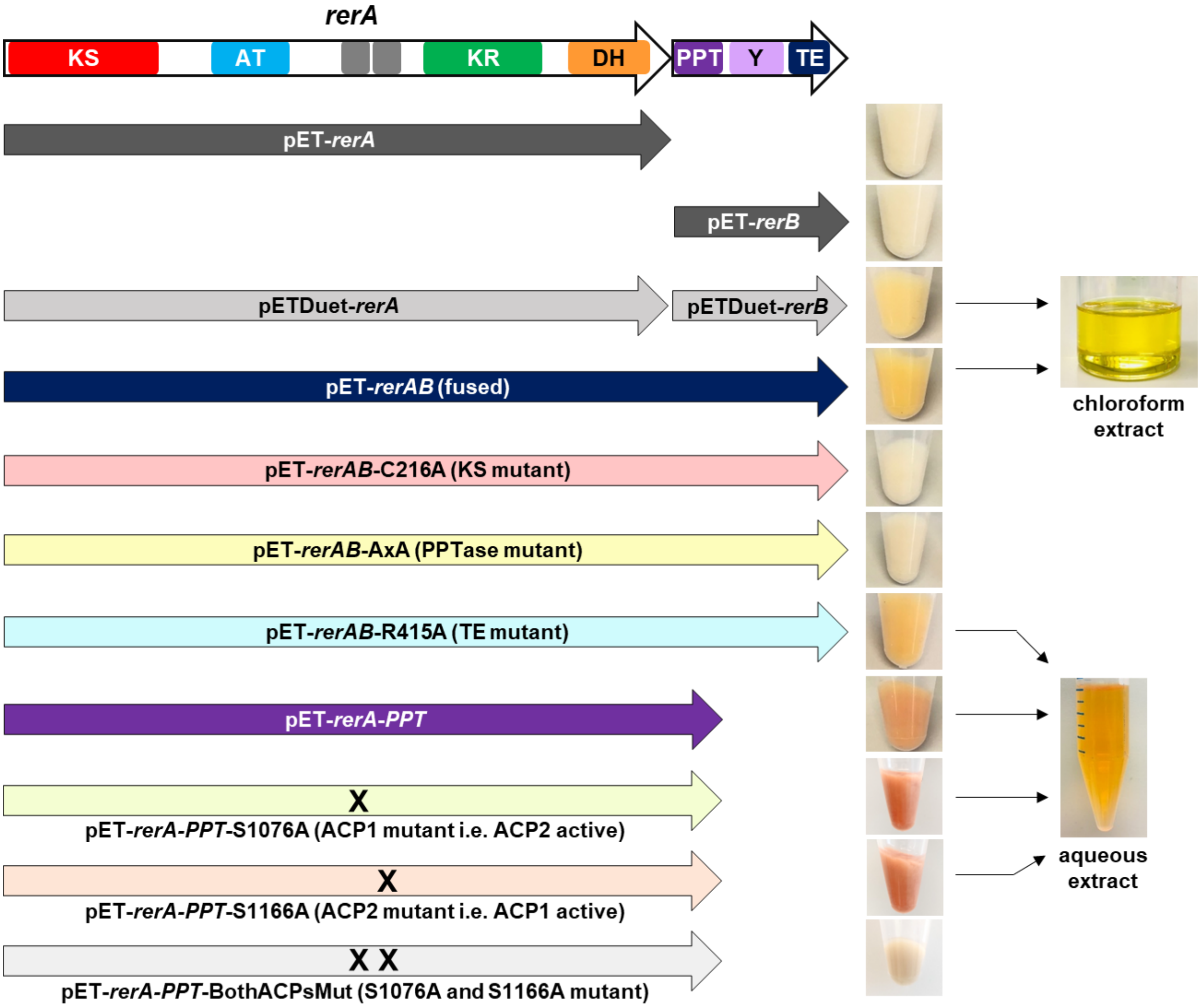

Figure S4 Illustration of all constructs generated for the *in vivo* expression of *rerA-B* in *E. coli* and the corresponding cell pellet and extracts colouration when expressed in *E. coli* BL21(DE3).

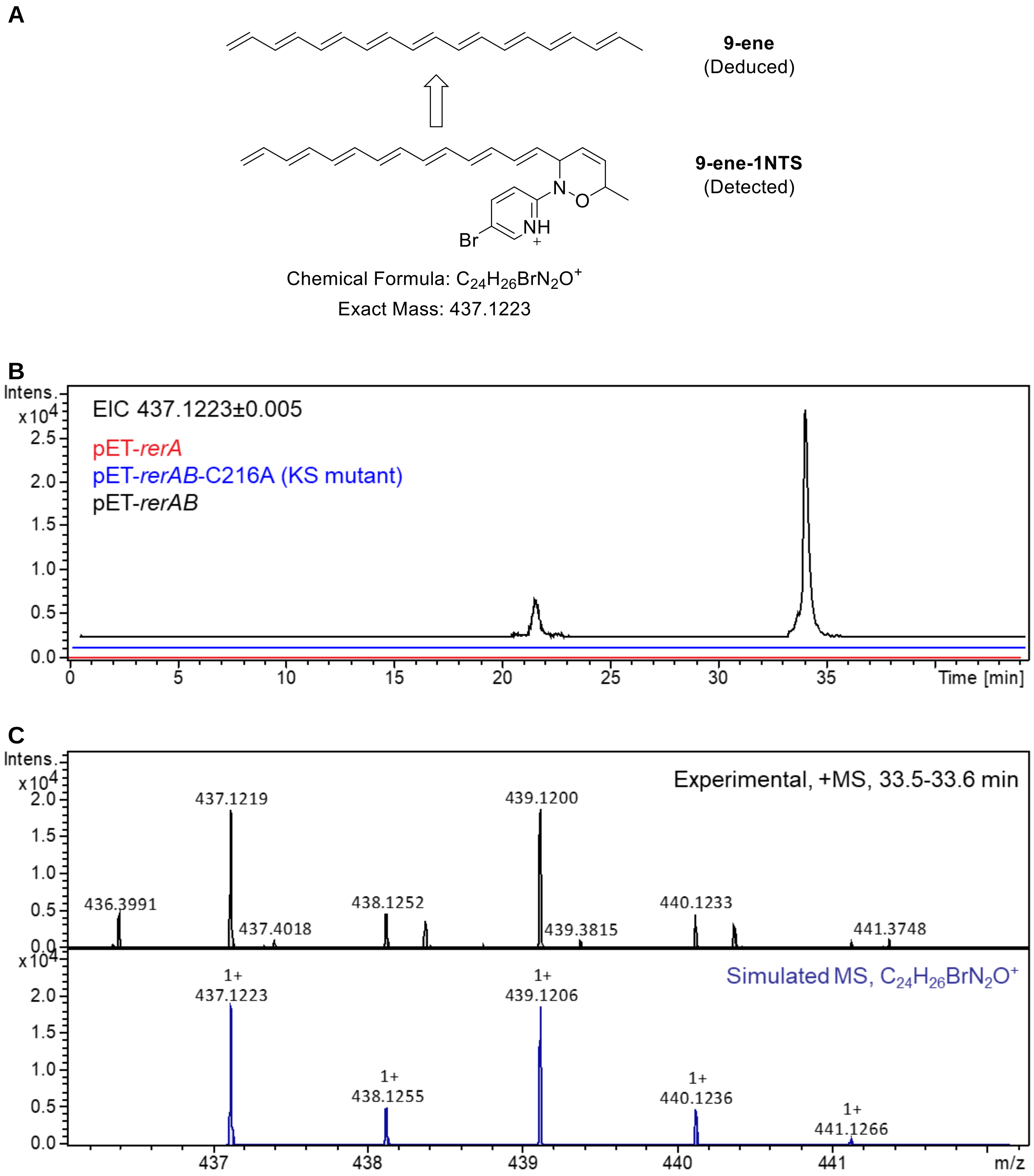

Figure S5 (A) Proposed structure of double NTS-derivatised nonaene (9-ene-1NTS). (B) Extracted Ion Chromatogram (EIC) of the monoisotopic mass of 9-ene-1NTS. (C) ESI Mass spectrum (top panel) along with its simulated isotopic distribution (bottom panel, simulated using DataAnalysis software version 4.4).

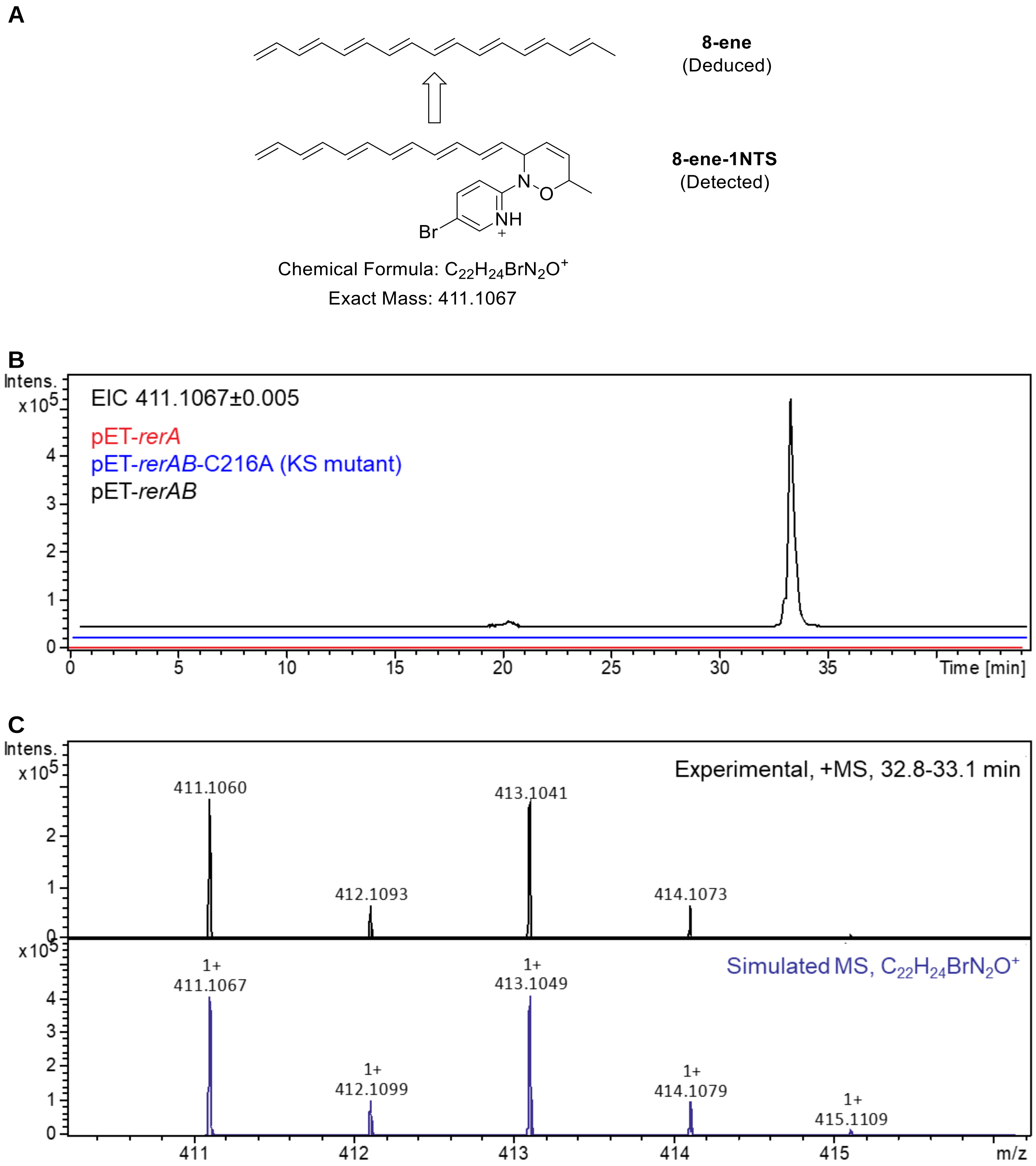

Figure S6 (A) Proposed structure of single NTS-derivatised octaene (8-ene-1NTS). (B) EIC of the monoisotopic mass of 8-ene-1NTS. (C) ESI-Mass spectrum (top panel) along with its simulated isotopic distribution (bottom panel).

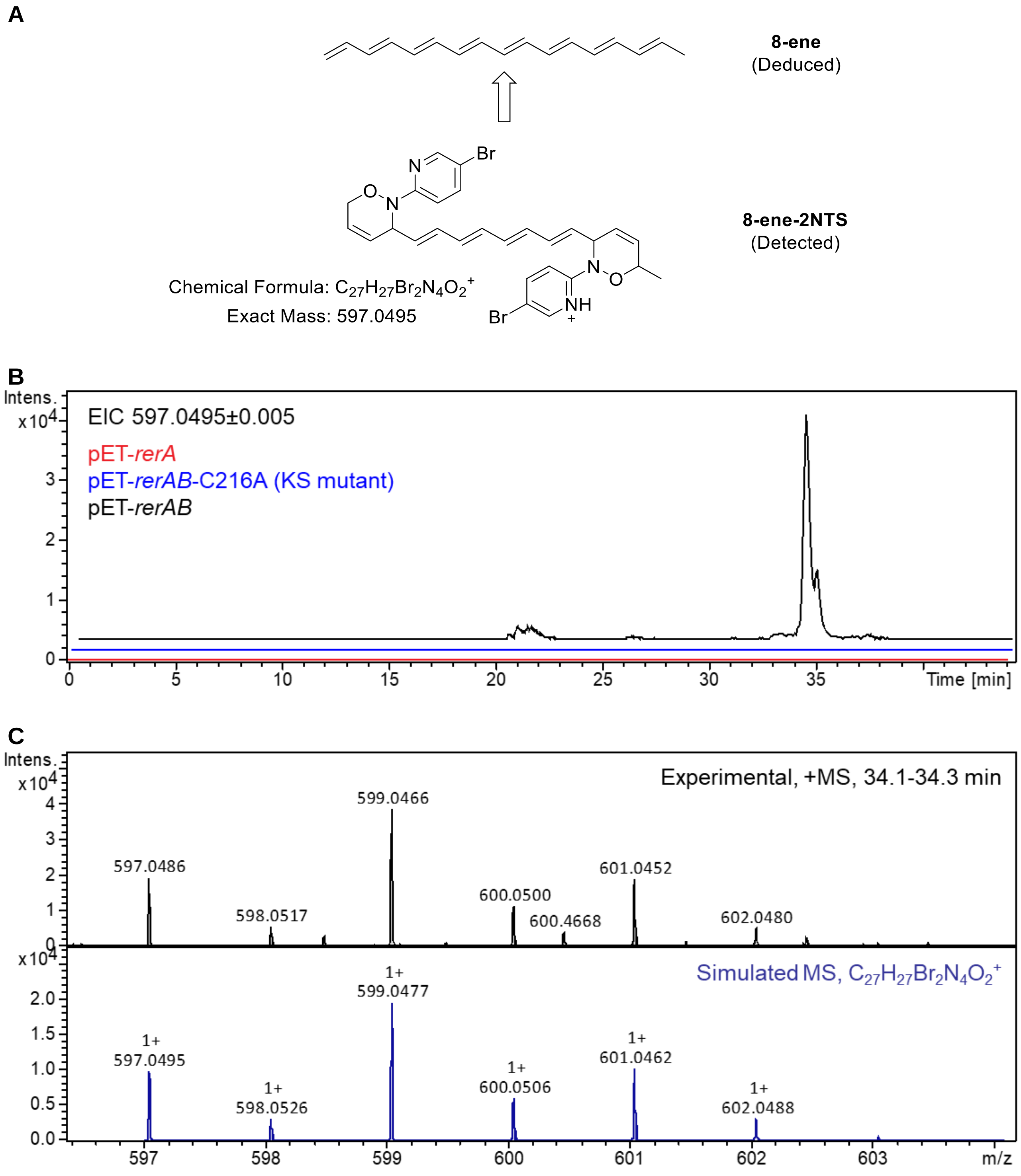

Figure S7 (A) Proposed structure of double NTS-derivatised octaene (8-ene-2NTS). (B) EIC of the monoisotopic mass of 8-ene-2NTS. (C) Mass spectrum (top panel) along with its simulated isotopic distribution (bottom panel).

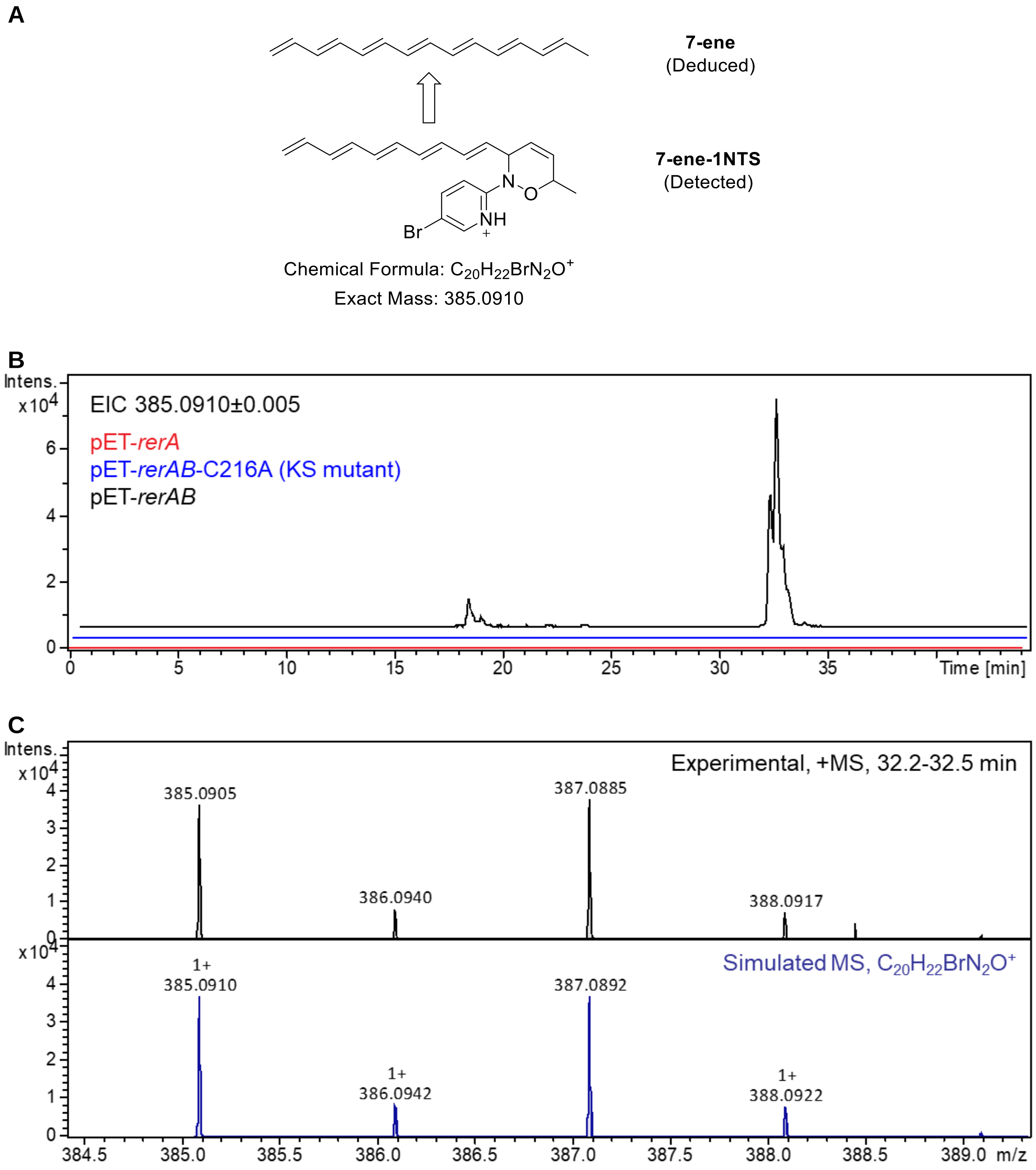

Figure S8 (A) Proposed structure of single NTS-derivatised heptaene (7-ene-1NTS). (B) EIC of the monoisotopic mass of 7-ene-1NTS. (C) Mass spectrum (top panel) along with its simulated isotopic distribution (bottom panel).

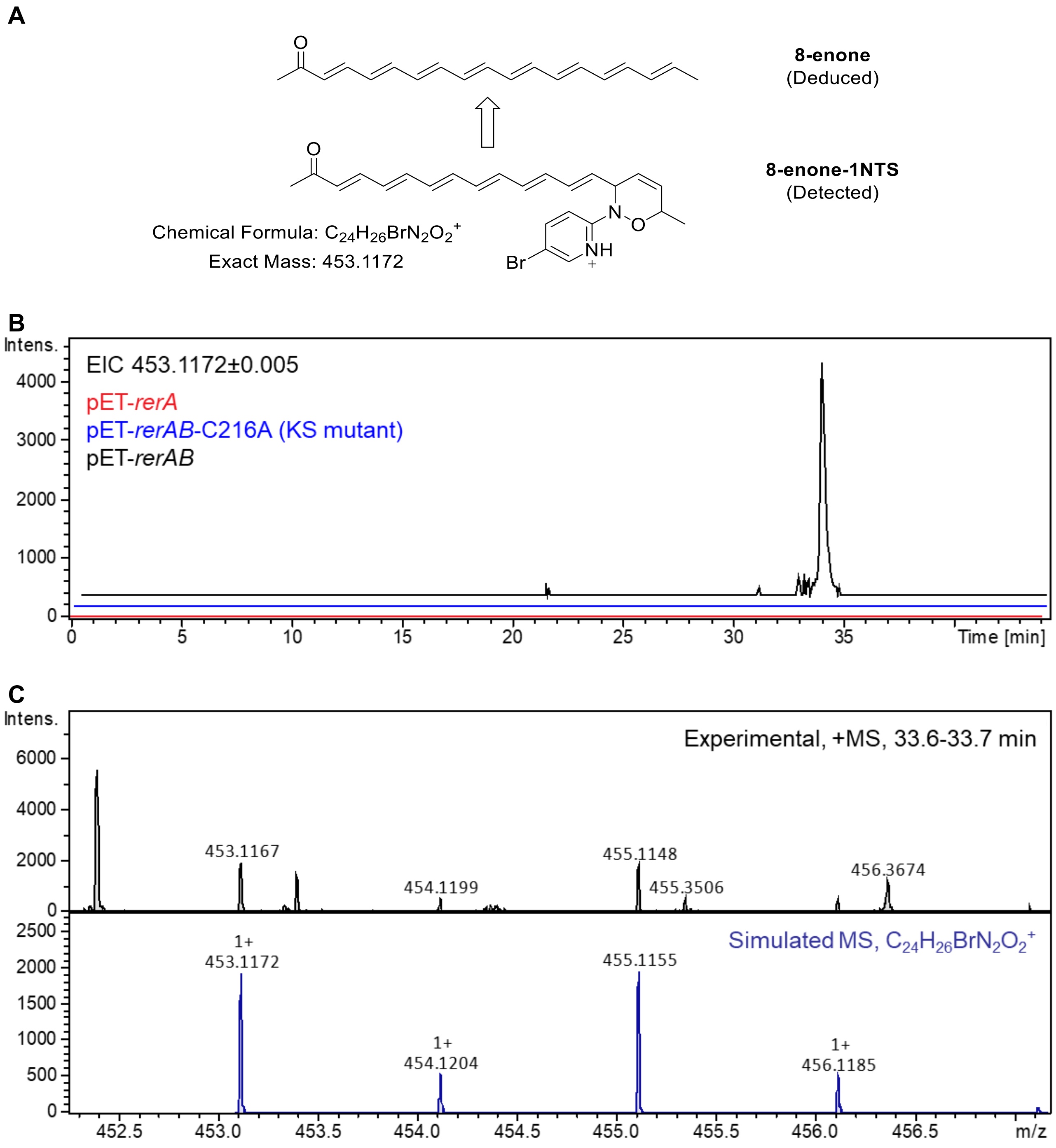

Figure S9 (A) Proposed structure of single NTS-derivatised octaenone (8-enone-1NTS). (B) EIC of the monoisotopic mass of 8-enone-1NTS. (C) Mass spectrum (top panel) along with its simulated isotopic distribution (bottom panel).

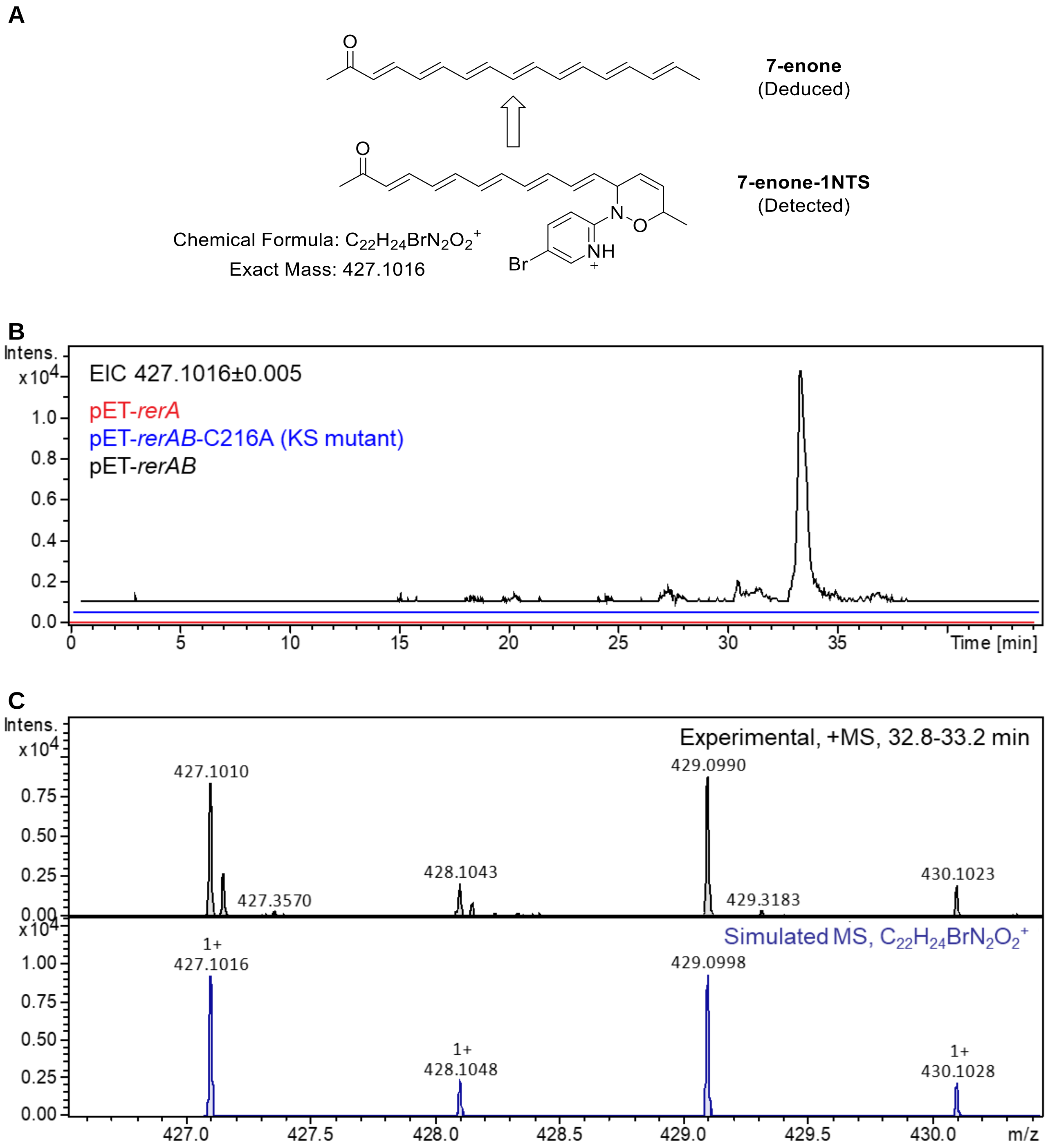

Figure S10 (A) Proposed structure of single NTS-derivatised heptaenone (7-enone-1NTS). (B) EIC of the monoisotopic mass of 7-enone-1NTS. (C) Mass spectrum (top panel) along with its simulated isotopic distribution (bottom panel).

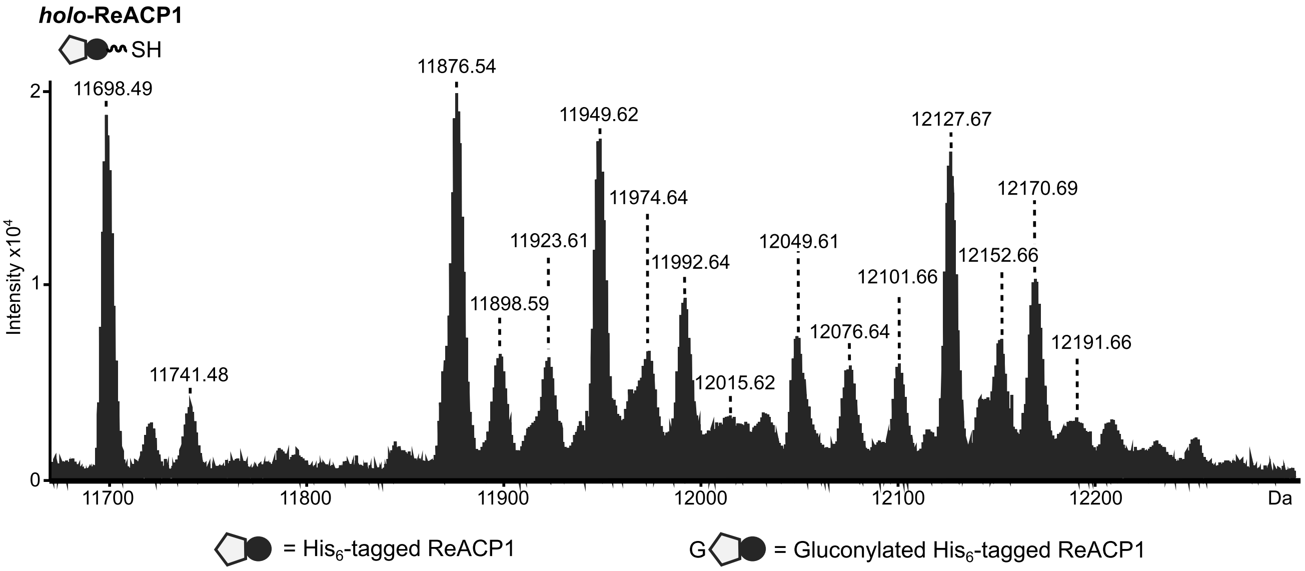

Mass detected (Da)	Proposed intermediate intercepted	Mass detected (Da)	Proposed intermediate intercepted
11698.49	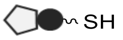	12015.62	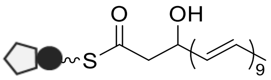
11741.48	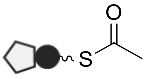	12049.61	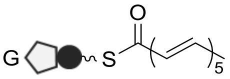
11876.54	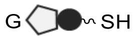	12076.64	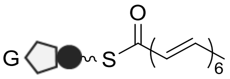
11898.59	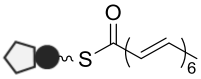	12101.66	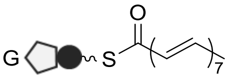
11923.61	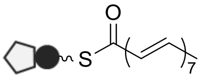	12127.67	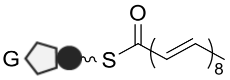
11949.62	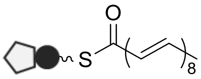	12152.66	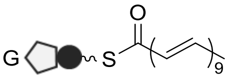
11974.64	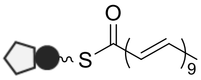	12170.69	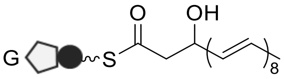
11992.64	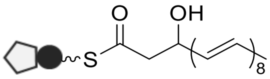	12191.66	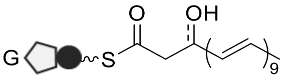

Figure S11 Deconvoluted complete mass spectrum of intact recombinant His6-ReACP1 purified from protein coexpression experiments showing different chain length polyketide/polyene intermediates bound to the ACP domain. G indicates gluconoylation of recombinant proteins with related mass increase of 178 Da. Deconvoluted spectrum for intact recombinant His6-ReACP2 purified from similar protein coexpression experiments showing different chain length polyketide/polyene intermediates bound to the protein available on request.

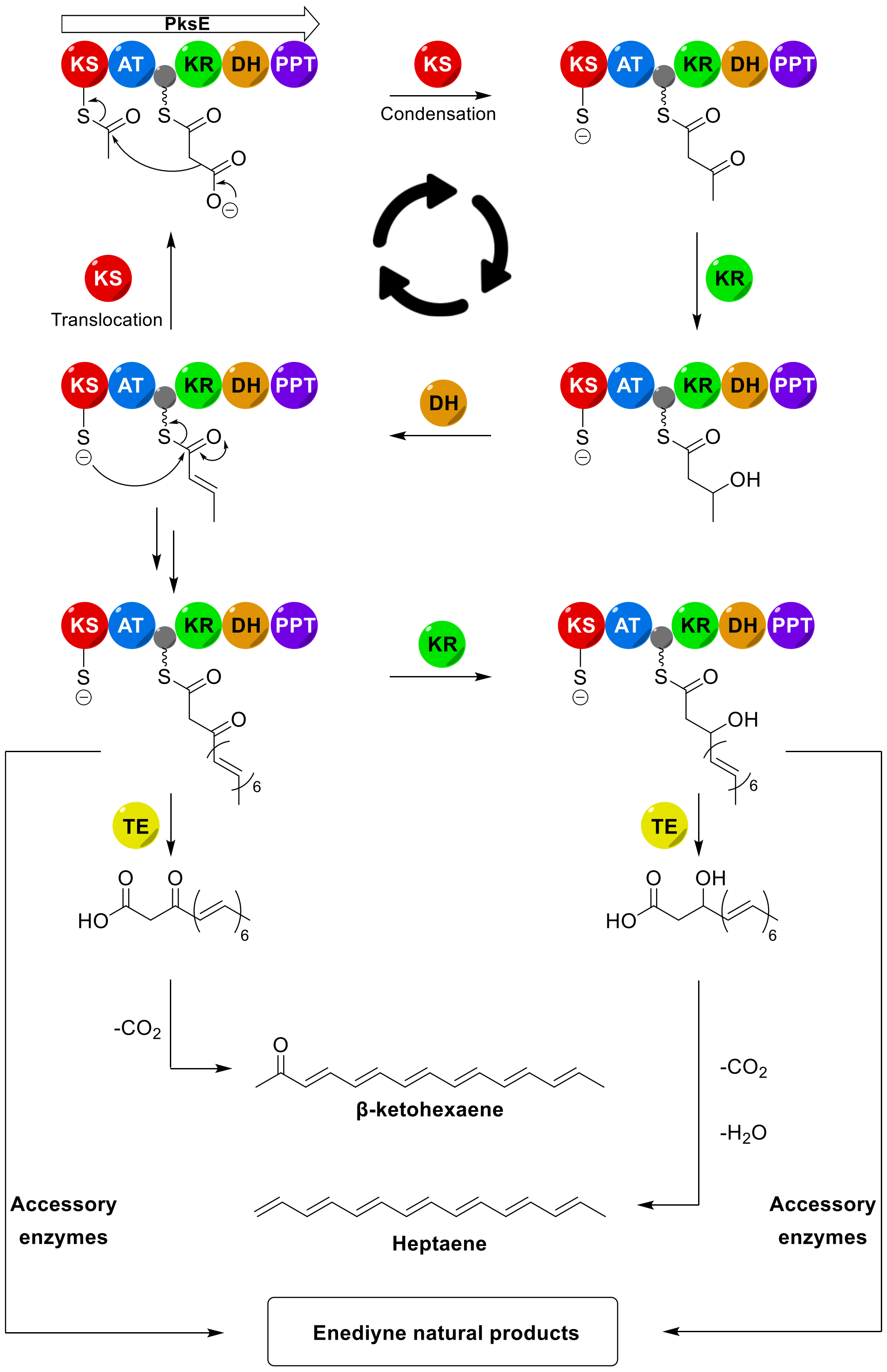

Figure S12 Current proposed biosynthesis of polyene molecules by an enediyne type I iPKS and its cognate TE, forming β-ketohexaene and heptaene shunt products (references 20 and 21 in main text). The mechanism of polyketide chain release from RerA-B is likely occurring in a similar way, based on the nature of the intermediates and products characterised (previous figures).

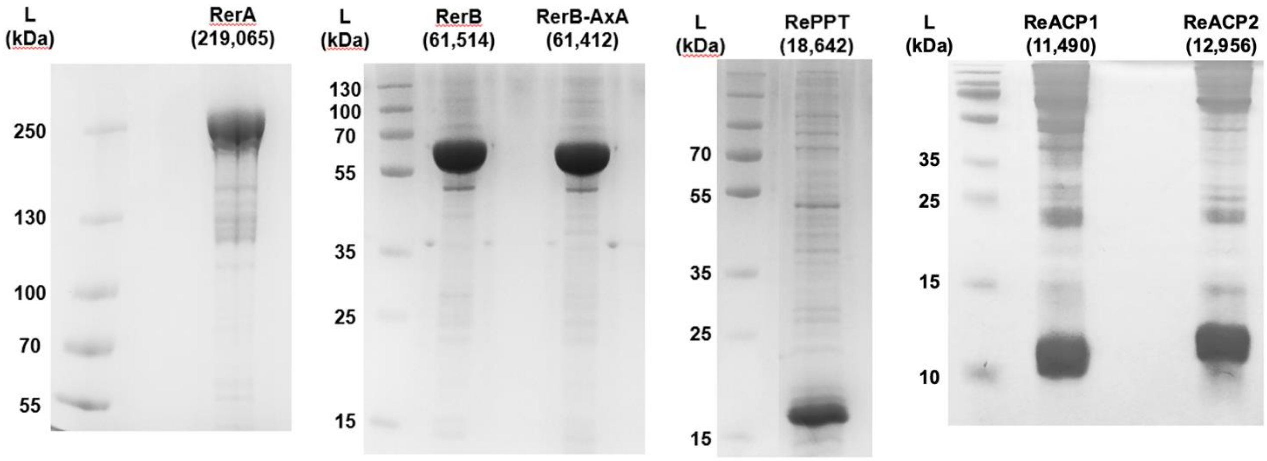

Figure S13 SDS-PAGE of purified recombinant proteins generated for iPKS activity reconstitution experiments (original uncropped gels shown in Figure S21).

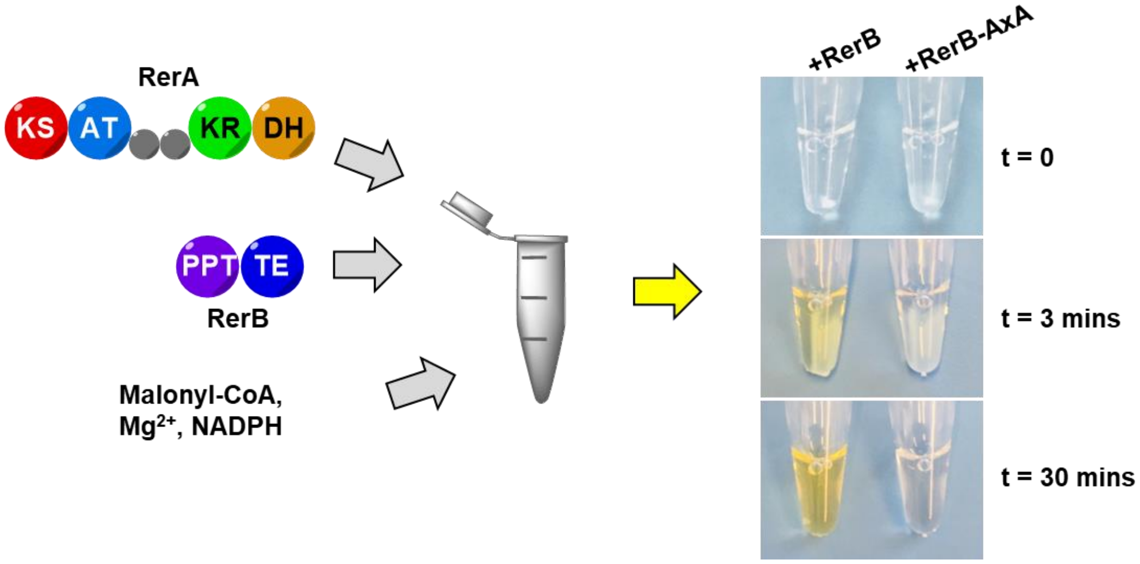

Figure S14 Illustration of *in vitro* reconstitution of RerA-B activity and time snapshots of reactions showing visible yellow colour development for active recombinant enzymes. RerB is a wild-type enzyme whereas RerB-AxA contains a double inactivation mutation in the binding sites (D48A and E50A) of the PPTase domain within RerB.

A

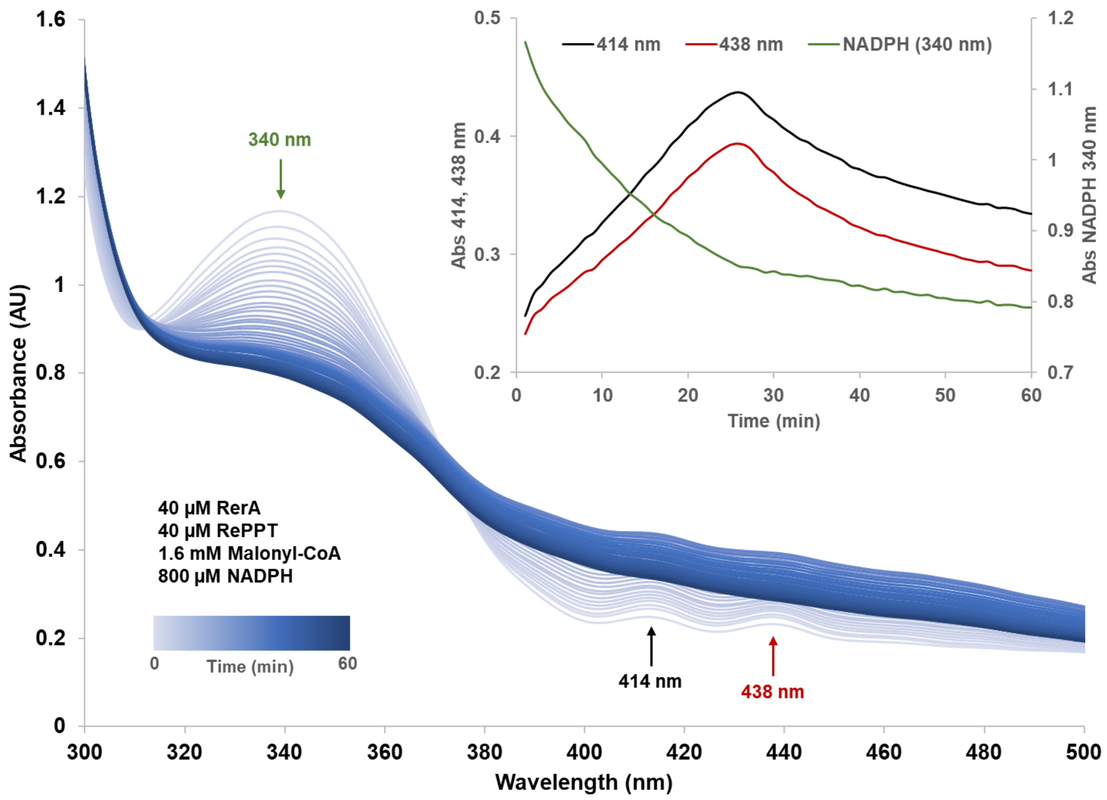

340 nm
414 nm
438 nm
40 μM RerA
40 μM RePPT
1.6 mM Malonyl-CoA
800 μM NADPH
Time (min)
0
60
Absorbance (AU)
Wavelength (nm)
414 nm
438 nm
NADPH (340 nm)
Abs 414, 438 nm
Abs NADPH 340 nm
Time (min)

B

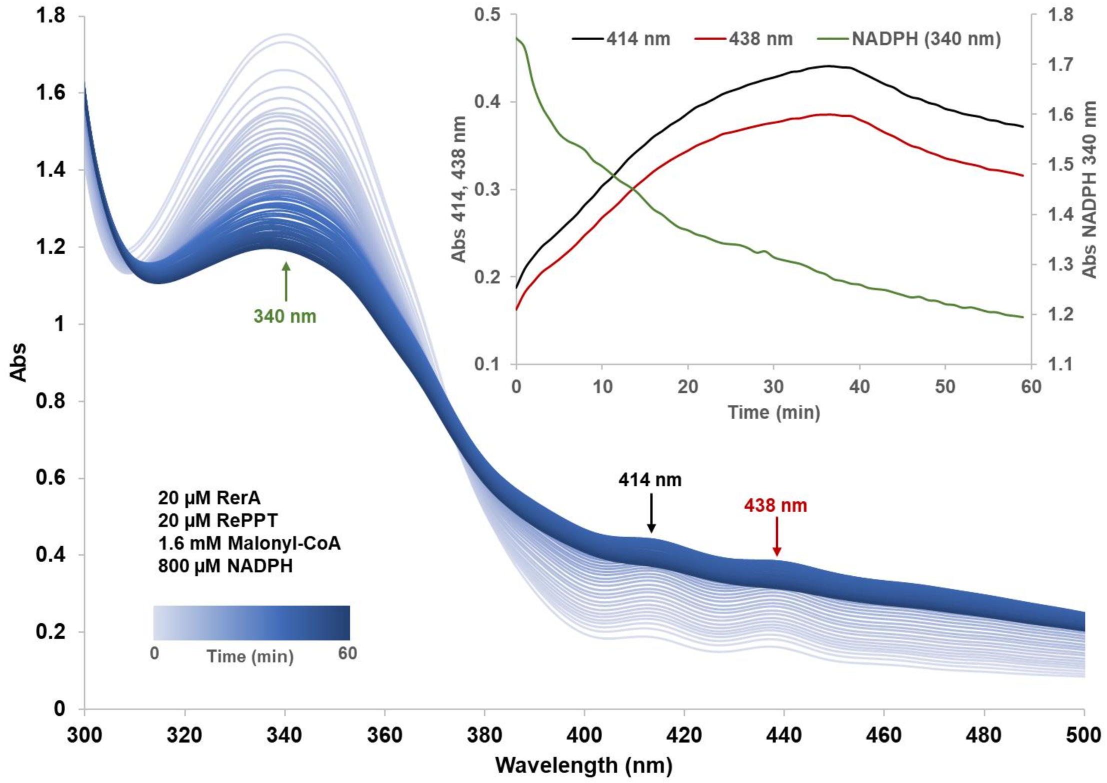

340 nm
414 nm
438 nm
20 μM RerA
20 μM RePPT
1.6 mM Malonyl-CoA
800 μM NADPH
Time (min)
0
60
Abs
Wavelength (nm)
414 nm
438 nm
NADPH (340 nm)
Abs 414, 438 nm
Abs NADPH 340 nm
Time (min)

C

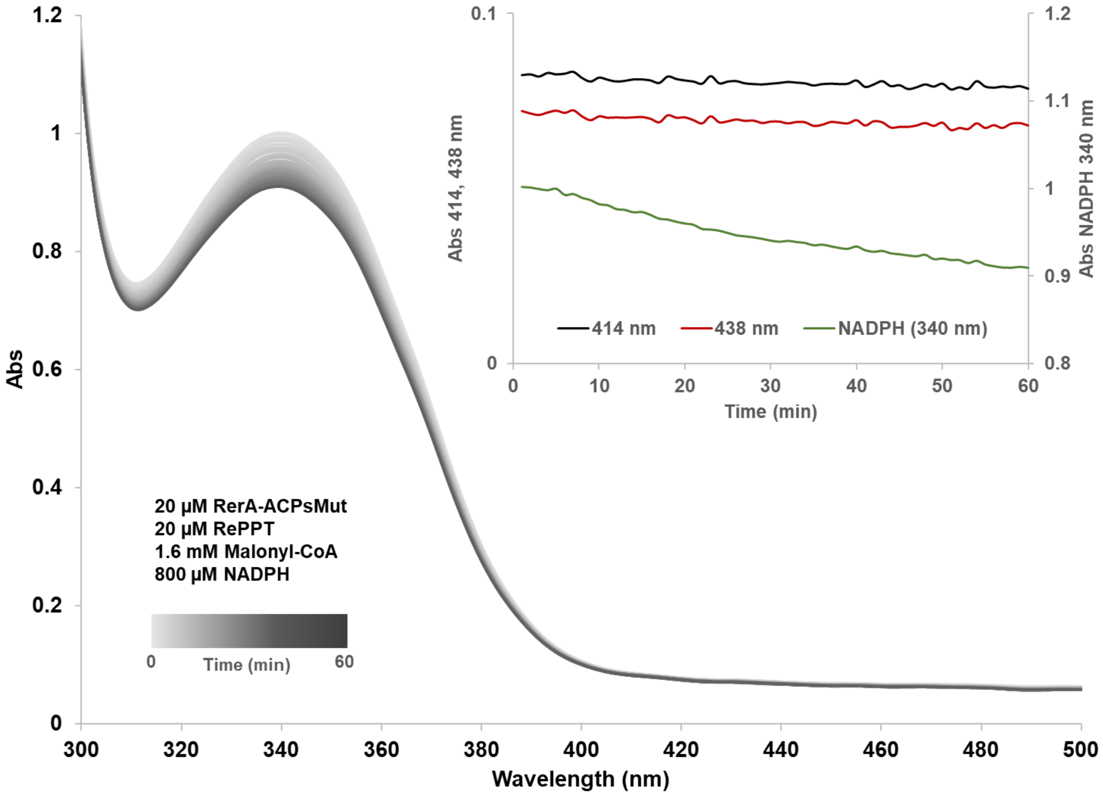

D

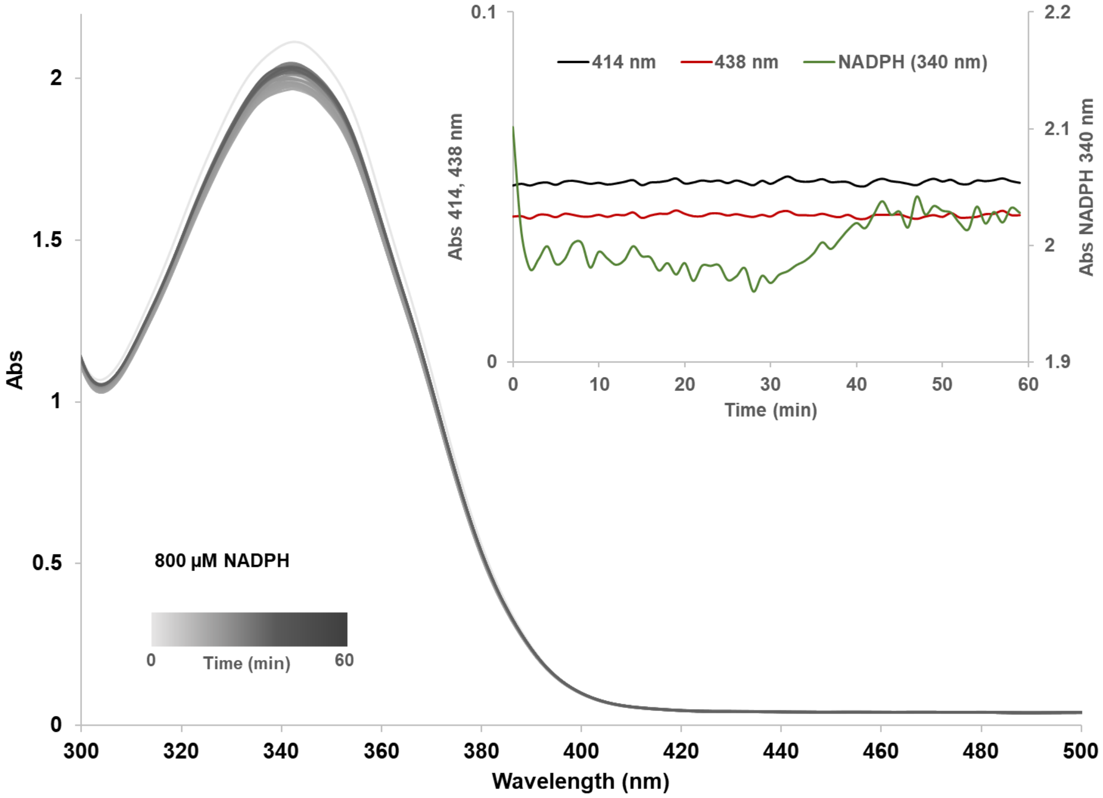

Figure S15 UV-Vis monitored time-course of *in vitro* reconstitution of RerA-B activity showing consumption of NADPH cofactor (340 nm) and generation of putative polyene products (as detectable by λ_{max} of 414 and 438 nm) in the insets. (A) Assay employing enzymes at 40 µM concentration; (B) assay employing enzymes at 20 µM concentration; (C) assay employing inactive RerA (RerA-ACPsMut protein); and (D) absorbance of NADPH cofactor in the reaction buffer.

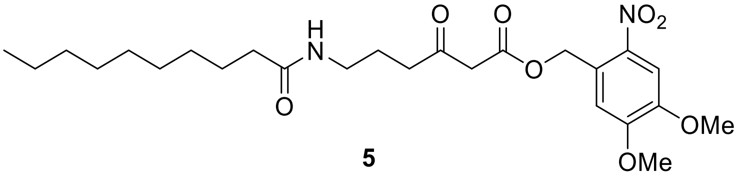

5

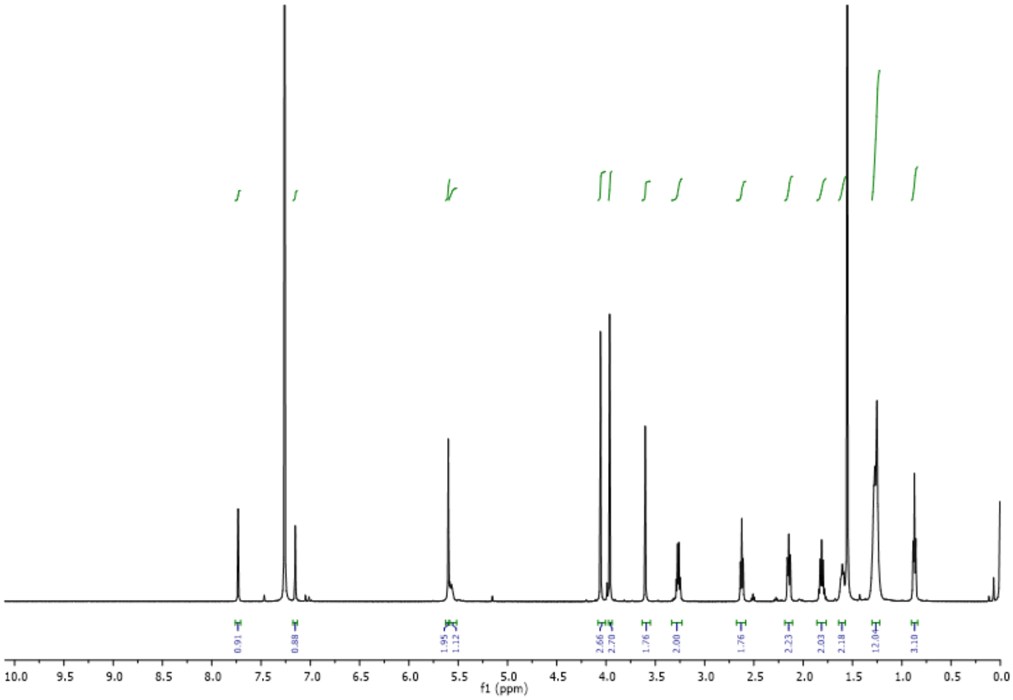

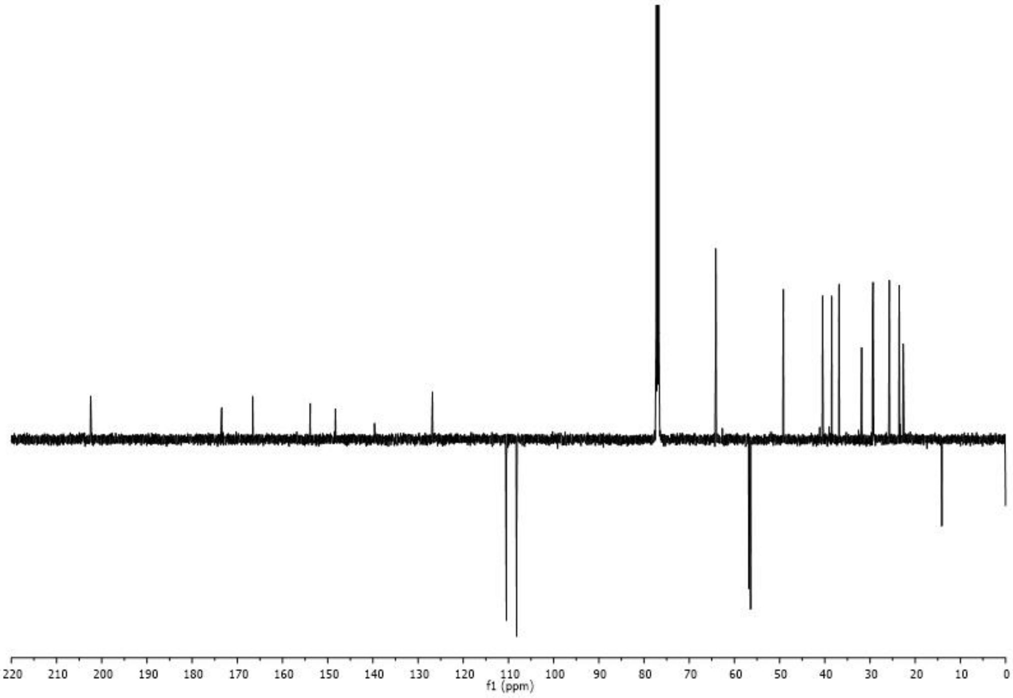

Figure S16 ^{1}H- and ^{13}C-NMR spectra (in $CDCl_3$) for photolabile probe **5**

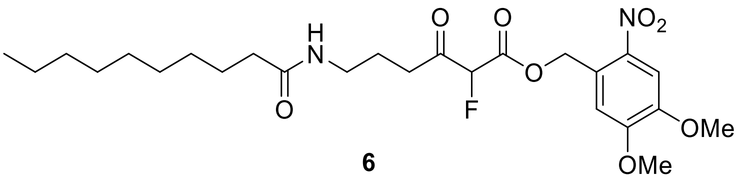

6

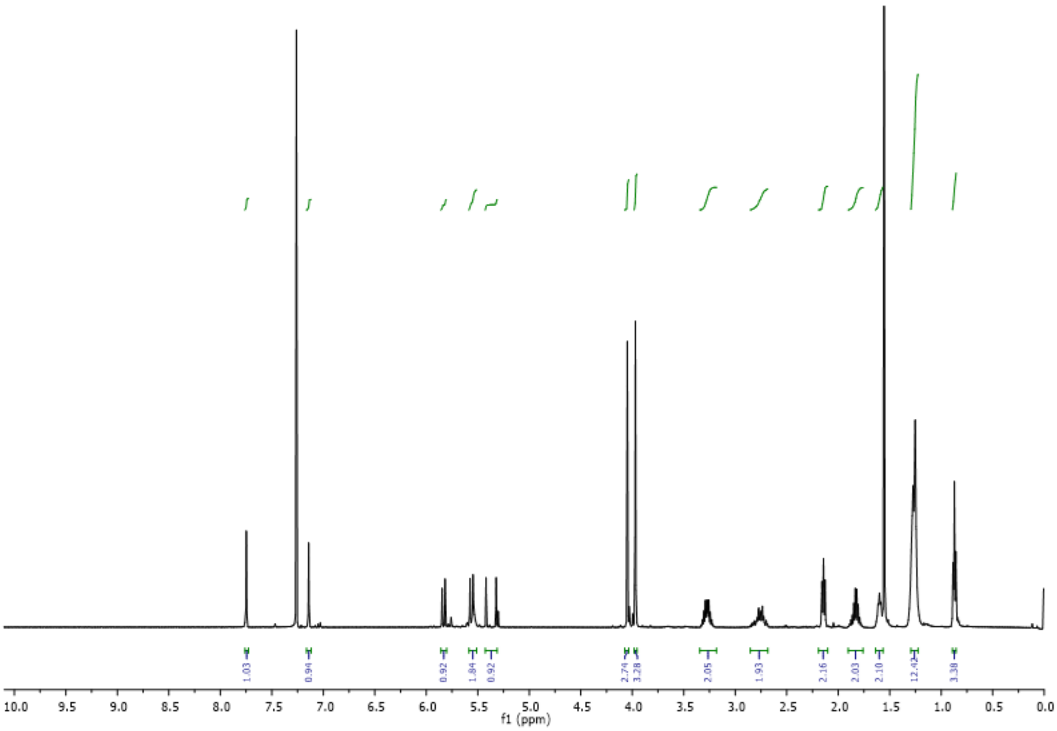

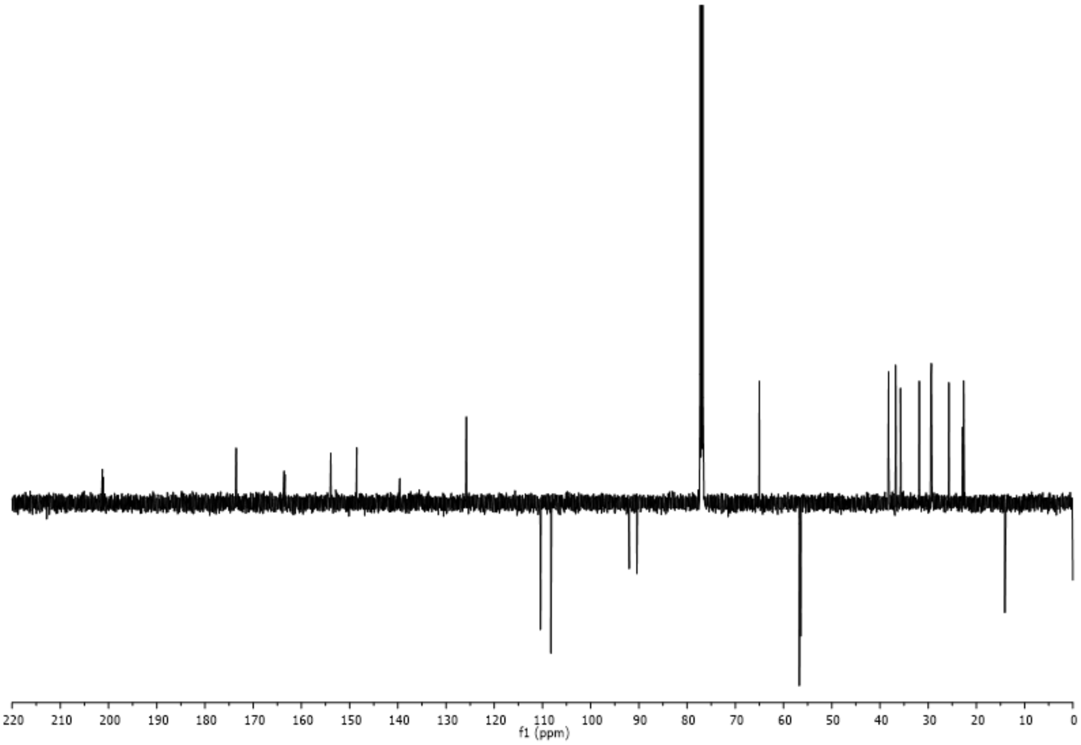

Figure S17 ^1H- and ^{13}C-NMR spectra (in $CDCl_3$) for photolabile probe **6**

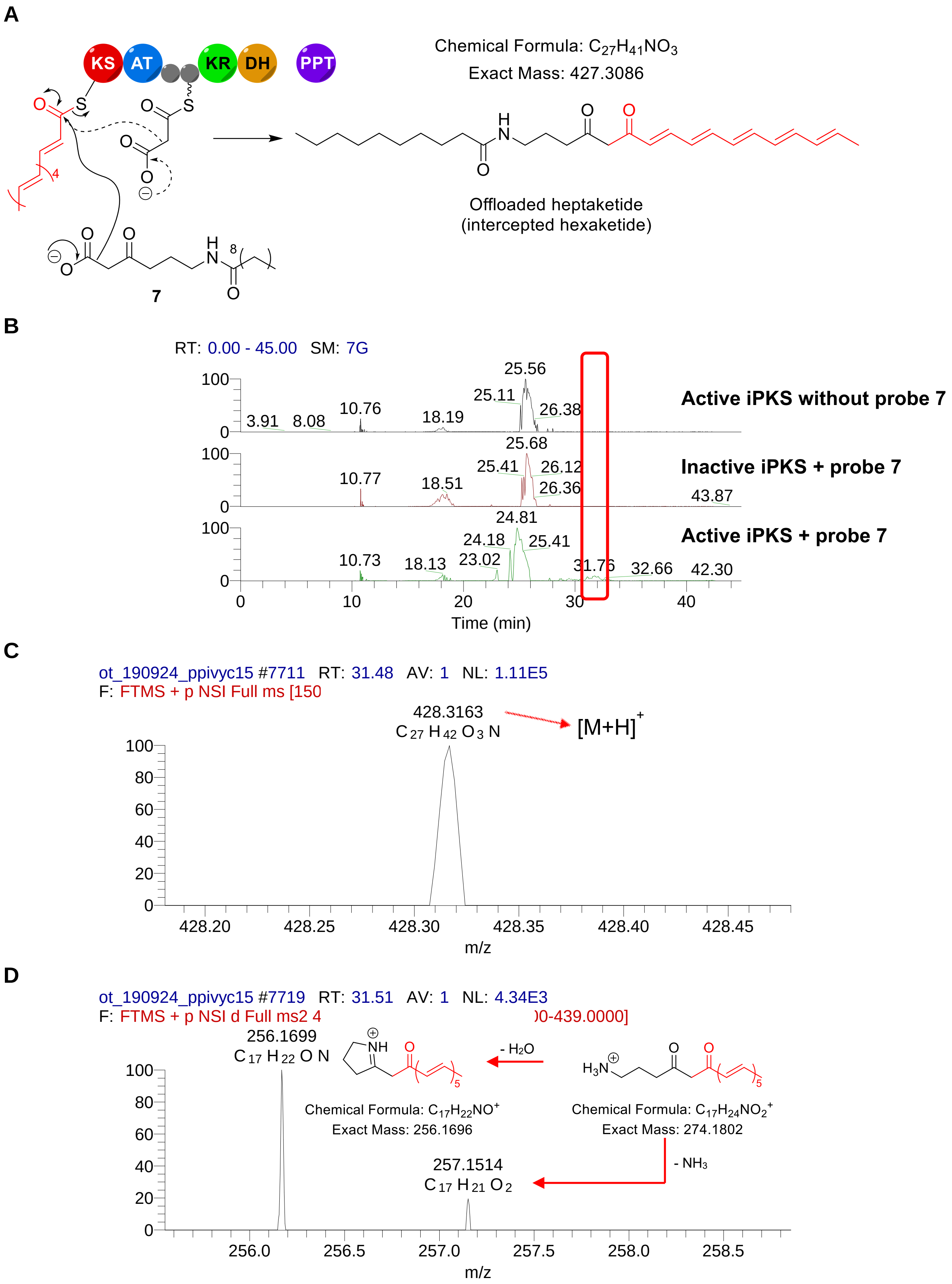

Figure S18 (A) Structure of putative heptaketide intercepted by probe **7**. (B) EIC of offloaded heptaketide. (C) Mass spectrum of offloaded heptaketide. (D) Diagnostic MS^2 fragmentation of offloaded heptaketide.

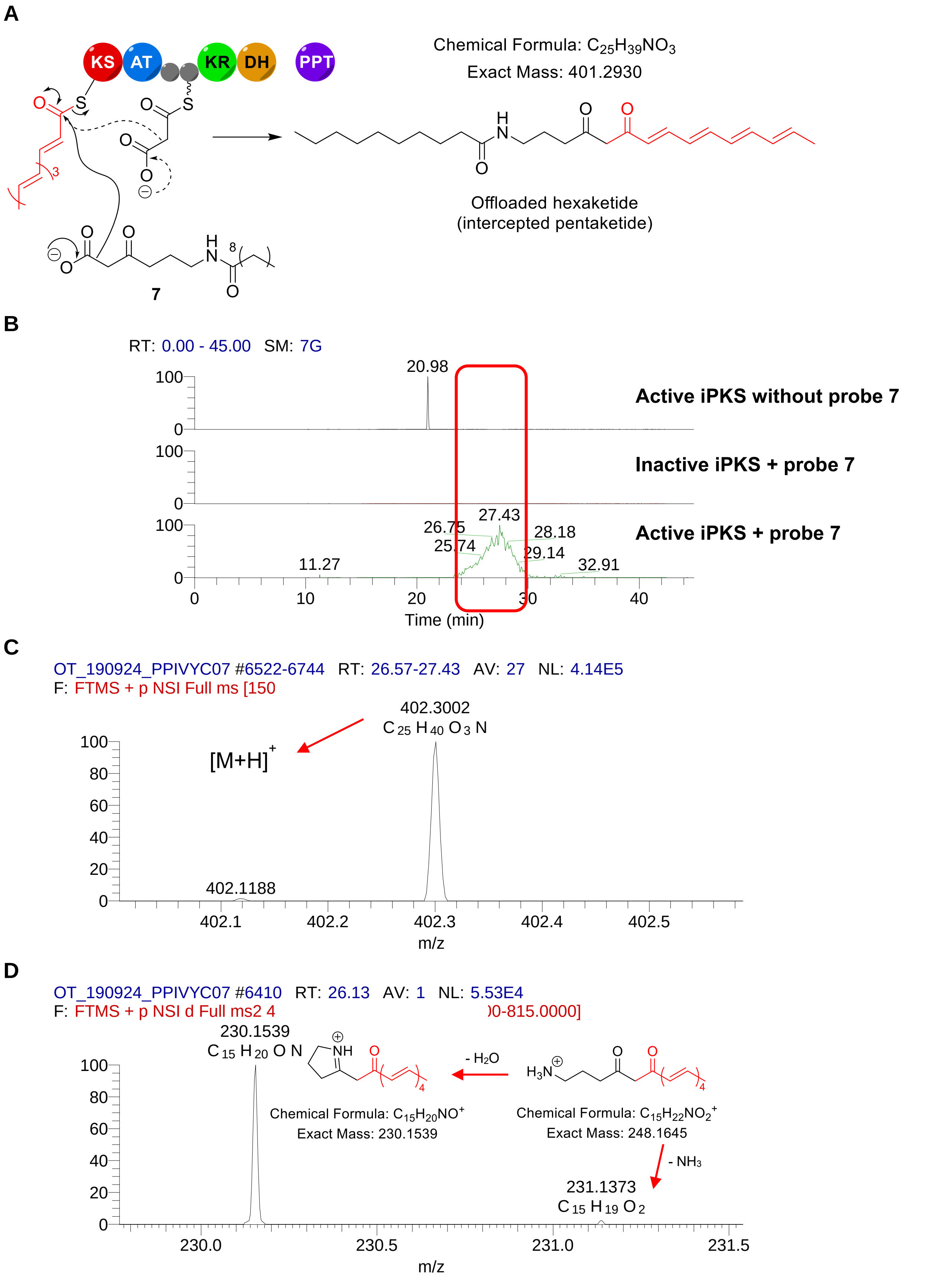

Figure S19 (A) Structure of putative hexaketide intercepted by probe **7**. (B) EIC of offloaded hexaketide. (C) Mass spectrum of offloaded hexaketide. (D) Diagnostic MS[2] fragmentation of offloaded hexaketide.

Detection and characterisation data of other intercepted species available on request.

Supplementary Tables

Table S1 AntiSMASH analysis of *Rhodococcus erythropolis* PR4 genome (NCBI accession no. NC_012490.1, antiSMASH version 7.1.0, date of analysis 25/07/2024). BGC stands for Biosynthetic Gene Cluster.

**BGC #**	**Type descriptor**	**Nucleotide position**		**Most similar known cluster (% similarity)**
		From	To	
1	Linear azol(in)e-containing peptides	113171	143275	Diisonitrile SF2768 (11%)
2	Type I PKS	233553	278496	-
3	Type I PKS	398638	444757	Fulvuthiacenes (8%)
4	Non-alpha poly-amino acids like ε-poly-lysine	596684	630577	ε-Poly-L-lysine (100%)
5	NRPS	1055114	1113305	Erythrochelin (57%)
6	Redox cofactor	1918747	1941562	Tetronasin (3%)
7	NRPS	1957712	2052893	Corynecins (100%)
8	NRPS-like	2501139	2543673	Thiolutin (8%)
9	NRPS	2868228	2926165	Heterobactins (100%)
10	NRPS, Terpene	3257161	3311461	SF2575 (6%)
11	NRPS	3342469	3409045	Coelichelin (27%)
12	NRPS	3657585	3724431	Rifamorpholine (4%)
13	NRPS	3772211	3828404	Monensin (5%)
14	Terpene	3907729	3928673	Carotenoid (27%)
15	Ectoine	4128539	4138937	Ectoine (75%)
16	Butyrolactone	5848307	5859194	-
17	PKS-like, Aminoglycoside	5866604	5907632	Acarbose (7%)
18	Lanthipeptide	6071308	6093890	-
19	Bacteriocin	6463713	6475643	Branched-chain fatty acid (75%)

Table S2 List of genes from BGC no. 3 and their proposed protein function

Locus tag	Putative function
RER_RS 03765	Hypothetical protein
RER_RS 33370	Hypothetical protein
RER_RS 32670	YbaB/EbfC family nucleoid-associated protein
RER_RS 03785	Aminotransferase class I/II-fold pyridoxal phosphate dependent enzyme
RER_RS 03790	DUF2029 domain-containing protein
RER_RS 03795	(Fe-S)-cluster assembly protein
RER_RS 03800	LysR family transcriptional regulator
RER_RS 03805	Putative sulfate exporter family transporter
RER_RS 03810	AraC family transcriptional regulator
RER_RS 03815	Quinone oxidoreductase
RER_RS 03820	TauD/TfdA family dioxygenase
RER_RS 03825	3-Keto-5-aminohexanoate cleavage protein
RER_RS 03830	MFS transporter
RER_RS 03835	Alpha/beta hydrolase
RER_RS 03840	Long chain fatty acid CoA ligase
RER_RS 03845	Carboxymuconolactone decarboxylase family protein
RER_RS 03850	Ammonium transporter
RER_RS 03855	DNA polymerase III subunit gamma and tau
**RER_RS 03860**	iPKS (KS-AT-ACP1-ACP2-KR-DH)
**RER_RS 03865**	PPTase-Y-TE
RER_RS 03870	Multicopper oxidase domain-containing protein
RER_RS 03875	Acetyl-CoA acetyltransferase
RER_RS 03880	Enoyl-CoA hydratase/isomerase family protein
RER_RS 03885	Hypothetical protein
RER_RS 03890	SAM-dependent methyltransferase
RER_RS 03895	FAD-binding oxidoreductase
RER_RS 32675	Hypothetical protein
RER_RS 03900	Polyketide cyclase/dehydratase
RER_RS 03905	Hypothetical protein
RER_RS 03910	Hypothetical protein
RER_RS 03915	N-acetylmuramoyl-L-alanine amidase
RER_RS 03920	YbaB/EbfC family nucleoid-associated protein
RER_RS 03925	Recombination protein RecR
RER_RS 03930	TetR family transcriptional regulator
RER_RS 03935	MFS transporter
RER_RS 03940	TetR/AcrR family transcriptional regulator
RER_RS 03945	MaoC family dehydratase
RER_RS 03950	OB-fold domain-containing protein
RER_RS 03955	MaoC family dehydratase

Table S3 A summary of NTS-derivatised polyene species detected by LC-MS from three independent experiments. A tick mark (✓) denotes detection at intensity >10^4 counts, whereas (?) denotes detection at intensity <10^4 counts.

**n**		**5**	**6**	**7**	**8**	**9**
n-ene	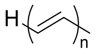	-	?	✓	?	-
n-ene-1NTS		?	?	✓	✓	✓
n-ene-2NTS		-	-	✓	✓	?
n-ene-3NTS		-	-	✓	✓	-
n-enone	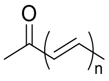	-	-	-	-	-
n-enone-1NTS		-	?	✓	?	-

Chemistry general methods and materials

Unless otherwise specified, all reactions were performed in oven-dried round bottom flasks in anhydrous solvents under argon. Chemicals and solvents were purchased from Sigma Aldrich, Fisher Scientific and VWR International (AR grade) and were used without any further purification. Analytical thin-layer chromatography was performed on aluminium sheets pre-coated with silica gel 60 (F254, Merck) using the indicated solvents, the spots were visualized under ultra-violet light (short wavelength) and using a stain of potassium permanganate, anisaldehyde, ninhydrin or vanillin, followed by heating using a heat gun. Silica column chromatography was carried out for purification with silica gel which was purchased from Sigma Aldrich (Tech Grade, pore size 60 Å, 230-400 mesh).

Compound analysis and characterisation

IR spectra were recorded as neat samples or thin films on a Bruker Alpha-T FTIR spectrometer with 24 scans. ^{1}H-, ^{13}C- and ^{19}F- NMR spectra were recorded on Bruker Avance instruments, including DPX-300 MHz, DPX-400 MHz and DPX-500 MHz. NMR samples were prepared in $CDCl_3$, CD_3OD, d^6-DMSO or D_2O unless otherwise stated. All coupling constant (J) values are quoted in Hertz (Hz) and are rounded to the nearest 0.1 for carbon couplings and 0.01 for proton couplings; all chemical shifts in parts per million (ppm). For ^{1}H- and ^{13}C- NMR data, the chemical shifts were reported relative to the solvent signal, such as $CDCl_3$ (δ_H 7.26 ppm, δ_C 77.2 ppm). High-resolution mass spectra (HR-MS) of novel compounds were obtained using electrospray ionisation (ESI) on a MaXis UHR-TOF (Bruker Daltonics) or on Bruker MaXis (ESI-HR-MS).

Synthesis of the nitrosopyridine (NTS) probe 3

This was carried out as previously reported by Castro-Falcón et *al.*[1]

Synthesis and characterisation of photolabile chemical probes

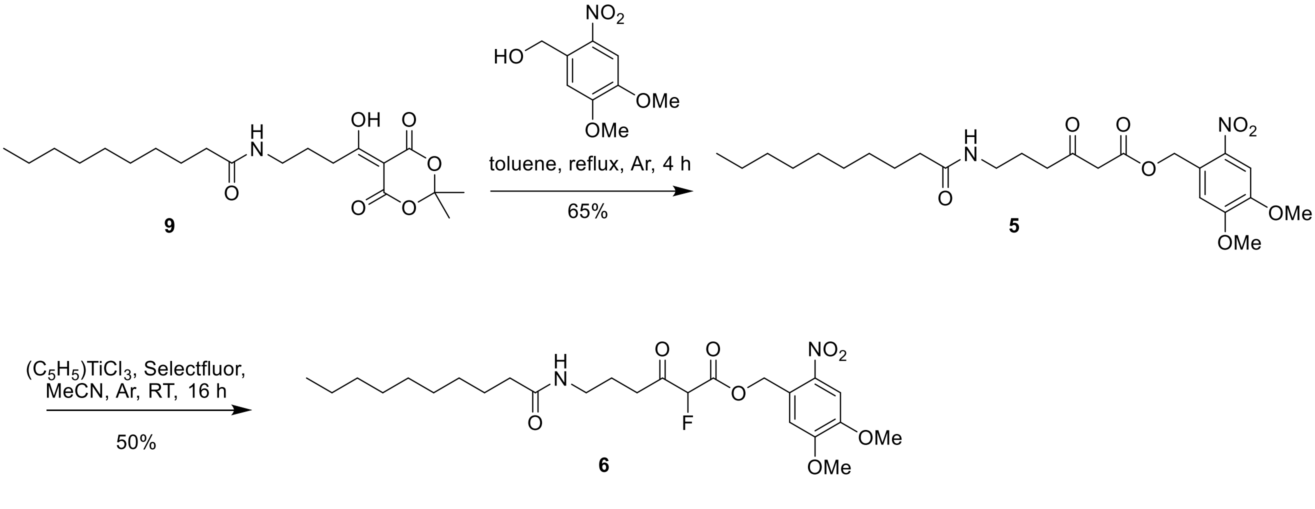

Scheme S1 Preparation of DMNB-protected chemical probes **5** and **6** from precursor **9** (previously reported).[2,3]

Synthesis of probe 5

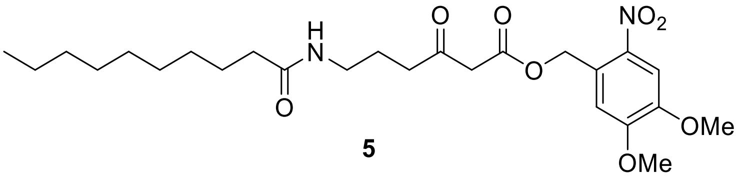

5

A stirring solution of *N*-(4-(2,2-dimethyl-4,6-dioxo-1,3-dioxan-5-ylidene)-4-hydroxybutyl) decanamide (**9**, 0.10 g, 0.26 mmol) and 4,5-dimethoxy-2-nitrobenzyl alcohol (0.07 g, 0.31 mmol) in anhydrous toluene (5 mL) was refluxed for 4 h under argon, then concentrated. Purification of the crude product by silica gel chromatography with a gradient of 20-90% EtOAc in petroleum ether afforded the title compound as a white solid (0.08 g, 65%). **TLC** (R_f = 0.40, 3:1 EtOAc : petroleum ether); **^{1}H NMR** (500 MHz, $CDCl_3$): δ 7.76 (1H, s, Ar*H*), 7.18 (1H, s, Ar*H*), 5.62 (2H, s, OCH_2Ar), 5.59-5.55 (1H, b m, N*H*), 4.08 (3H, s, OCH_3), 3.99 (3H, s, OCH_3), 3.63 (2H, s, COCH_2CO), 3.29 (2H, q, J = 6.6, CH_2), 2.65 (2H, t, J = 6.9, CH_2), 2.17 (2H, t, J = 7.6, CH_2), 1.84 (2H, quin, J = 7.2, CH_2), 1.63 (2H, quin, J = 7.0, CH_2), 1.60-1.55 (10H, m, CH_2), 1.30-1.28 (3H, m, CH_3); **^{13}C NMR** (125 MHz, $CDCl_3$): 202.5 (CH_2COCH_2), 173.5 (R*C*ONH), 166.6 (RCO_2R'), 153.9 (NO_2CCCH*C*OCH3), 148.3 (NO2CCH*C*OCH3), 139.6 (NO_2*C*), 126.8 (NO_2C*C*), 110.5 (NO_2*C*H), 108.2 (NO_2C*C*H), 64.1 (CO_2CH_2C), 56.8 (H_3*C*OCCHC), 56.4 (H_3*C*OCCHCNO_2), 49.2 (CO_2CH_2CO), 40.5 (CONHCH_2), 38.5 (CO_2CH_2COCH_2), 36.9 (CH_2CONH), 31.9 ($CH_2CH_2COCH_2$), 29.5 (CH_2), 29.4 (CH_2), 29.3 (CH_2), 29.3 (CH_2), 25.8 (CH_2), 23.6 (CH_2), 22.7 (CH_2CH_3), 14.1 (CH_3); IR (thin film) υmax = 3326, 2921, 2846, 1709, 1750, 1214, 1630, 1271, 1517, 1325 cm^{-1}; HRMS (ESI): calculated for $C_{25}H_{37}N_2O_8$ $[M-H]^-$ 493.2551, found: 493.2555.

Synthesis of probe 6

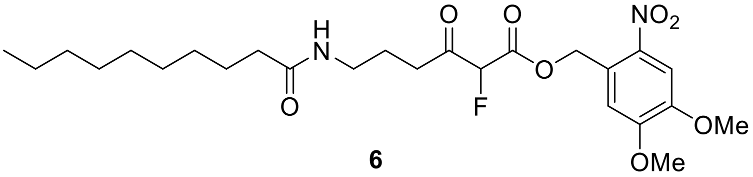

6

To a stirring solution of 4,5-dimethoxy-2-nitrobenzyl 6-decanamido-3-oxohexanoate (**5**, 50 mg, 0.10 mmol) and cyclopentadienyltitanium trichloride (1 mg, 0.01 mmol) in anhydrous MeCN (1 mL) under argon, Selectfluor (38 mg, 0.11 mmol) was added.[4] After 16 h stirring, the reaction was concentrated, diluted with H_2O (4 mL) and extracted with EtOAc (4 x 4 mL). The combined organic extracts were washed with brine (10 mL), dried over $MgSO_4$(s), filtered, and concentrated to the crude product. Purification by silica gel chromatography with a gradient of 0-40% EtOAc in petroleum ether afforded the title compound as a white solid (26 mg, 50%): **TLC** (Rf = 0.38, 1:1 petroleum ether: EtOAc); **^{1}H NMR** (500 MHz, $CDCl_3$): δ 7.75 (1H, s, ArC*H*), 7.14 (1H, s, ArC*H*), 5.83 (1H, d, J = 14.8, CO_2CH_2), 5.56 (1H, d, J = 14.8, CO_2CH_2), 5.53 (1H, br s, N*H*), 5.37 (1H, d, J = 48.9, C*H*F), 4.05 (3H, s, OCH_3), 3.97 (3H, s, OCH_3), 3.27 (2H, qq, J = 13.6, 6.62, NHCH_2), 2.86-2.68 (2H, m, $CH_2COCH_2CO_2$), 2.14 (2H, t, J = 8.0, CH_2CONH), 1.83 (2H, tq, J = 14.0, 6.9, NHCH_2CH_2), 1.64-1.57 (2H, m, CH_2CONH), 1.33-1.21 (12H, m, CH_2), 0.87 (3H, t, J = 6.9, CH_2CH_3); **^{13}C NMR** (125 MHz, $CDCl_3$): 201.3 (d, J = 23.0, CH_2COCH_2), 173.7 (*C*ONH), 163.6 (d, J = 23.7, CO_2), 154.1 (NO_2CCH*C*), 148.7 (OCH_2CC*C*OCH_3), 139.7 (OCH_2*C*CCOCH_3), 125.9 (NO_2*C*), 110.6 (OCH_2CC*C*OCH_3), 108.4 (NO_2C*C*H), 91.4 (d, J = 198.8, *C*HF), 65.2 (CO_2CH_2), 56.9 (H_3*C*OCCHC), 56.6 (H_3*C*OCCHCNO_2), 38.4 (NHCH_2), 36.9 (CH_2CONH), 35.9 (CH_2COCHF), 32.0 (CH_2), 29.6 (CH_2), 29.5 (CH_2), 29.5 (CH_2), 29.4 (CH_2), 25.9 (CH_2CH_2CONH), 23.0 (NH CH_2CH_2), 22.8 (CH_2), 14.0 (CH_3); **^{19}F NMR**: -113.2 (*C*FH); **IR** (thin film) υmax = 3301, 2924, 2853, 1765, 1733, 1644, 1522, 1276, 1221, 1066 cm^{-1}; HRMS (ESI): calculated for $C_{25}H_{37}FN_2NaO_8$ $[M+Na]^+$: 535.2426, found: 535.2424.

General methods and materials for molecular biology

All chemicals including antibiotics were purchased from either Merck (UK) or Thermo Fisher Scientific (UK). Primers (oligonucleotides) were synthesised by Merck (UK). Q5 High-Fidelity DNA Polymerase and NEBuilder HiFi DNA Assembly Master Mix were purchased from New England Biolabs (NEB UK). All restriction enzymes and ligases were purchased from NEB. All enzymes were stored at -20 °C and used with the buffers provided and at suggested temperatures.

List of plasmid vectors used

Plasmid	Description	Source
pET28a	Kan^R, PT7, pBR322 *ori*, N-terminal His_6-tag, thrombin cleavage site, for protein expression	Novagen
p28TEV	Kan^R, PT7, pBR322 *ori*, N-terminal His_6-tag, TEV cleavage site, for protein expression	Tosin group
pUC19	Amp^R, pMB1 *ori*	Invitrogen
pETDuet-1	Amp^R, PT7, pBR322 *ori*, for protein coexpression	Novagen
pACYCDuet-1	Cm^R, PT7, p15A *ori*, for protein coexpression	Novagen

Bioinformatic tools

AntiSMASH[5]: https://antismash.secondarymetabolites.org/
NCBI search: https://blast.ncbi.nlm.nih.gov/Blast.cgi
Clustal Omega[6]: https://www.ebi.ac.uk/jdispatcher/msa/clustalo
ESPript[7]: https://espript.ibcp.fr/ESPript/ESPript/
Cblaster[8]: https://cagecat.bioinformatics.nl/tools/search

Accession number

Microorganism name	NCBI accession number
Rhodococcus erythropolis PR4	NC_012490.1

List of plasmids generated

All plasmids were assembled *via* one of the following four methods: restriction digestion and ligation (L), Gibson assembly (G), TOPO cloning (T), or mutagenesis of existing plasmid (M).

Plasmid name	Tag	Resistance marker	Primers	Assembly method	Insertion sites	Insert length (bp)	Protein mass (Da)	pI
p28T-rerA	N-His	Kan	P1 + P2	L	*Nde*I-*Not*I	6120	219,064.90	4.97
pET28a-rerB	N-His	Kan	P3 + P4	L	*BamH*I-*Not*I	1587	61,513.78	5.10
pET28a-rerB-AxA	N-His	Kan	P5 + P6	M	*BamH*I-*Not*I	1587	61,411.73	5.18
pET28a-rerB-R415A	N-His	Kan	P7 + P8	M	*BamH*I-*Not*I	1587	61,428.67	5.06
pETDuet-RerB	C-S-tag	Amp	P9 + P10	G	*Nde*I-*Xho*I	1584	60,204.28	4.85
pETDuet-RerAB	N-His	Amp	P11 + P12	L	*Asc*I-*Not*I	6120	218,987.79	4.97
p28T-rerAB (fused)	N-His	Kan	P13 + P14, P15 + P16	G	*Nde*I-*Xho*I	7716	276,930.58	4.94
p28T-rerA-PPT	N-His	Kan	P13 + P17	G	*Nde*I-*Xho*I	6594	235,498.07	4.92
*pUC19-2307	-	Amp	-	L	*Nde*I-*Kpn*I	2307	-	-
*pUC19-2307-C216A	-	Amp	P18 + P19	M	*Nde*I-*Kpn*I	2307	-	-
p28T-rerAB-C216A (KS mutant)	N-His	Kan	-	L	*Nde*I-*Xho*I	7716	276,898.52	4.94
p28T-rerAB-AxA (PPT mutant)	N-His	Kan	P13 + P14, P15 + P16	G	*Nde*I-*Xho*I	7716	276,828.53	4.95
p28T-rerAB-R415A (TE mutant)	N-His	Kan	P13 + P14, P15 + P16	G	*Nde*I-*Xho*I	7716	276,845.47	4.93
p28T-rerA-PPT-S1076A (ACP1 mutant)	N-His	Kan	P13 + P20, P21 + P17	G	*Nde*I-*Xho*I	6594	235,482.07	4.92
p28T-rerA-PPT-S1166A (ACP2 mutant)	N-His	Kan	P13 + P22, P23 + P17	G	*Nde*I-*Xho*I	6594	235,482.07	4.92
p28T-rerA-PPT-BothACPsMut	N-His	Kan	P13, P24; P25, P17	G	*Nde*I-*Xho*I	6594	235,466.07	4.92
pACYC-rerA-PPT-BothACPsMut	-	Cm	P26 + P24, P25 + P27	G	*Nco*I-*Xho*I	6594	233,060.53	4.87
p28T-rerA-ACPsMut	N-His	Kan	P1 + P2	G	*Nde*I-*Not*I	6120	219,032.90	4.97

p28T-RePPT	N-His	Kan	P28 + P17	G	*Nde*I-*Xho*I	465	18,642.60	5.01
p28T-ReACP1	N-His	Kan	P29 + P30	G	*Nde*I-*Xho*I	264	11,490.51	4.80
p28T-ReACP2	N-His	Kan	P31 + P32	G	*Nde*I-*Not*I	246	12,956.36	5.02

*pUC19-2307 was an intermediate plasmid purposely generated to create C216A mutation in p28T-rerAB (fused) plasmid. Two restriction enzymes flanking the mutagenic site (*Nde*I and *Kpn*I) were identified in p28T-rerAB. pET-rerAB was then digested with *Nde*I and *Kpn*I restriction enzymes to generate a linear DNA of 2307 bp in size. This piece of DNA was ligated into pUC19 plasmid predigested with *Nde*I and *Kpn*I restriction enzymes, thus generating a small intermediate plasmid of 4765 bp named pUC19-2307. Site-directed mutagenesis (TGC → GCC; C216A) was carried out on this plasmid which resulted in a mutagenic plasmid named pUC19-2307-C216A. To generate p28T-rerAB-C216A, pUC19-2307-C216A was again digested with *Nde*I and *Kpn*I restriction enzymes, and the resulting linear mutagenic DNA was re-ligated back into the backbone of p28T-rerAB to yield desired p28T-rerAB-C216A plasmid.

List of primers used in this study

Code	Name	Sequence (5′ → 3′)
P1	RerA-NdeI-Fwd	CACC**CATATG**ACTGAAGATTCAACTAA
P2	RerA-NotI-Rev	ATTAT**GCGGCCGC**TCATTTGATCACCACATC
P3	RerB-BamHI-Fwd	TCGC**GGATCC**GGTGATCAAATGAGTAATGCCTTTGCCATC
P4	RerB-NotI-Rev	AGT**GCGGCCGC**TACTTTCCGTCGATCGCC
P5	RerB-AxAmut-Fwd	C**GCA**CCGATTCGCTCCCGAACC
P6	RerB-AxAmut-Rev	AT**TGC**ACACCCCTGAGGCCAGTC
P7	RerB-R415Amut-Fwd	GTCGAGGGTT**GCA**GACAAGTTCC
P8	RerB-R415mut-Rev	TGCCAGATGTAATAGTTG
P9	Duet-RerB-GA-Fwd	GTATAAGAAGGAGATATA**CATATG**AGTAATGCCTTTGCCATCG
P10	Duet-RerB-GA-Rev	GTTTCTTTACCAGA**CTCGAG**CTTTCCGTCGATCGCCTCC
P11	Duet-RerA-AscI-Fwd	AGCTC**GGCGCGCC**TGATGACTGAAGATTCAACTAA
P12	Duet-RerA-NotI-Rev	ATTAT**GCGGCCGC**TCATTTGATCACCACATC
P13	RerA-GA-NdeI-Fwd	CTGTATTTTCAGGGA**CATATG**ACTGAAGATTCAACTAATCCCG
P14	RerA-GSlink-Rev	TTACTACCGCTGCCGCTACCTTTGATCACCACATCCTCATCG
P15	RerB-GSlink-Fwd	TCAAAGGTAGCGGCAGCGGTAGTAATGCCTTTGCCATCGAAT
P16	RerB-GA-XhoI-Rev	TGGTGGTGGTGGTG**CTCGAG**CTACTTTCCGTCGATCGCCT
P17	PPT-GA-XhoI-Rev	TGGTGGTGGTG**CTCGAG**TCATGCGGCATCCGCCTCA
P18	C216Amut-Fwd	GGACGGTGCG**GCC**GCGTCCGGAATGC
P19	C216Amut-Rev	ACGACGTACCCGCCGCCG
P20	ACP1-S1076A-Rev	GCACTGAT**CGC**GTCGAGATTCAGATCATC
P21	ACP1-S1076A-Fwd	ATCTCGAC**GCG**ATCAGTGCGGCAGAGGCTATT
P22	ACP2-S1166A-Rev	CCTTGAT**CGC**GTCCATGTTCAGGTCGTCC
P23	ACP2-S1166A-Fwd	CATGGAC**GCG**ATCAAGGCCGCCGATGC
P24	PKS7-Rev	CAAGGATCAGCGATGCTGCT
P25	PKS7-Fwd	AGCAGCATCGCTGATCCTTG
P26	ACYC-RerA-Fwd	TAATAAGGAGATATACCATGACTGAAGATTCAACTAATCCCG
P27	ACYC-PPT-Rev	CGGTTTCTTTACCAGACTCGAGTCATGCGGCATCC
P28	RerB-GA-NdeI-Fwd	CTGTATTTTCAGGGA**CATATG**AGTAATGCCTTTGCCATCG
P29	ACP1-NdeI-Fwd	CTGTATTTTCAGGGA**CATATG**TCCGTGGAAAGTGAGCTG
P30	ACP1-XhoI-Rev	GGTGGTGGTGGTG**CTCGAG**CTACGCCGAACTCGCTG
P31	ACP2-NdeI-Fwd	CTGTATTTTCAGGGA**CATATG**TCGGACGTGTCGCGAATA
P32	ACP2-NotI-Rev	GTGCTCGAGT**GCGGCCGC**TATGATATACCCTTTGCCTTCGC

General cloning procedure

Target DNA was amplified from genomic DNA of *R. erythropolis* PR4 or other template by PCR using Q5 High-Fidelity DNA Polymerase (NEB) per manufacturer's protocol, analysed by agarose gel electrophoresis and subsequently purified using Monarch DNA gel extraction kit (NEB). Single or double restriction enzyme digestions were carried out according to the manufacturer's guidelines. The target fragments were then separated by agarose gel electrophoresis and subsequently purified. Ligation reactions were performed using Anza T4 DNA Ligase (Invitrogen) or by Gibson assembly method (NEBuilder HiFi DNA Assembly, NEB). An aliquot of assembled reactions was used for transformation of competent *E. coli* TOP10. Once single colonies could be observed, a single colony was inoculated into LB medium supplement with appropriate antibiotic (100 µg/mL for ampicillin, 50 µg/mL for kanamycin or 35 µg/mL for chloramphenicol) for the selection of uptaken plasmid.

Site-directed mutagenesis

Mutagenesis reactions were carried out using Q5 Site-Directed Mutagenesis Kit (NEB) in accordance with the manufacturer's recommended protocol. The primers were designed using NEBaseChanger website (http://nebasechanger.neb.com/). Once the PCR reaction was completed, the PCR product was treated with kinase, ligase and *Dpn*I enzymes (KLD enzyme mix) supplied with the kit. Thereafter, an aliquot was used to transform *E. coli* TOP10 for plasmid amplification.

Recombinant protein overproduction in *E. coli*

A single colony of transformed *E. coli* BL21(DE3) was selected, inoculated in 5 mL of LB medium containing an appropriate antibiotic and cultured overnight at 37 °C with agitation. The resulting preculture was added to 500 mL of LB medium with the same antibiotic and shaken at 37 °C and 180 rpm until its optical density at 600 nm reached a value between 0.6 – 0.8. Protein expression was induced by addition of 0.5 mM isopropyl-β-D-thiogalactoside (IPTG) then induced culture was shaken at 180 rpm overnight at 15 °C. The cells were then harvested by centrifugation (3,000 *g*, 20 min, 4 °C) and resuspended in 10 mL of Resuspension buffer (20 mM Tris, 100 mM NaCl, 20 mM imidazole, pH 7.5). Following resuspension, the cells were either stored at –80 °C or directly lysed for protein purification.

Protein purification

Cell pellet containing recombinant protein was lysed by sonication using Sonics Vibra-Cell VCX130 ultrasonic processor (settings: amplitude = 80%, pulse on = 10 s, pulse off = 30 s, time = 3 min), and the resulting lysate was centrifuged (21,000 *g*, 30 min, 4 °C). The clarified lysate was loaded onto a HisTrap HP 1 mL column pre-charged with 100 mM $NiSO_4$. Loosely bound and unbound proteins were washed off by passing Resuspension buffer (15 mL; 20 mM Tris, 100 mM NaCl, 20 mM imidazole, pH 7.5) through the column. Ni^{2+}-bounded His_6-tagged proteins were then eluted off the column using Low Imidazole Elution buffer (5 mL; 20 mM Tris, 100 mM NaCl, 50 mM imidazole, 10% glycerol (v/v), pH 7.5) followed by High Imidazole Elution buffer (15 mL; 20 mM Tris, 100 mM NaCl, 300 mM imidazole, 10% glycerol (v/v), pH 7.5). The presence of the recombinant protein was confirmed by SDS-PAGE analysis. Fractions containing the desired protein were combined, its buffer was exchanged for Protein Storage buffer (20 mM Tris, 100 mM NaCl, 10% glycerol (v/v), pH 7.5) and concentrated using a Vivaspin Centrifugal Concentrator of appropriate molecular weight cut-off. The concentrated protein was aliquoted, flash-frozen with liquid nitrogen and stored at –80 °C.

Extraction of yellow-coloured products

Cell pellets obtained from *E. coli* cultures expressing RerAB (from p28T-rerAB plasmid) were washed by resuspending in sterile water (20 mL per cell pellet from 1 L of culture), centrifuged (3,000 *g*, 10 min, 10 °C), and the supernatant was discarded. Water-washed cell pellets were lyophilised overnight using CoolSafe freeze dryer. Once dried, the residue was physically crushed into fine powder using mortar and pestle then transferred to a clean glass container. Chloroform (20 mL per lyophilised pellet obtained from 1 L of culture) was then added to the crushed material to extract yellow compounds. The crude extract was clarified by passing through a glass column fitted with cotton wool and Celite. The filtrate obtained was subsequently used for further analyses.

Extraction of orange protein solution from RerA-PPT and RerA-PPT-Y

Cell pellets obtained from *E. coli* cultures expressing RerA-PPT(-Y) (from p28T-rerA-PPT or p28T-rerA-PPT-Y plasmid) were washed twice by resuspending in sterile water (10 mL per pellet from 1 L of culture), centrifuged (3,000 *g*, 10 min, 10 °C), and the supernatant was discarded. After washing, the cell pellets were resuspended again in sterile water (10 mL per pellet from 1 L of culture) and lysed by sonication to obtain a bright orange solution. It is crucial to lyse the cell suspension as orange proteins cannot be sufficiently released from unlysed cells suspension. The cell lysate was centrifuged (21,000 *g*, 15 min, 4 °C) and the orange supernatant was directly used for further analyses.

Characterisation of coloured material by UV-Vis spectroscopy

UV-Vis absorption spectra were acquired using Varian Cary 50 UV-Vis spectrophotometer by scanning the sample in the range of 200 – 800 nm with scan rate of 300 nm/min. UV-Vis spectrum of chloroform extract from lyophilised RerAB is shown in Figure 2B (black trace) with absorption maxima detected at 448, 429, 403, 382, 362 and 344 nm; and UV-Vis spectrum of orange protein solution from RerA-PPT is shown in Figure 2B (red trace) with absorption maxima detected at 462, 438, 414, 390 and 366 nm.

In vitro reconstitution of core iPKS enzymes (RerA-B)

All in vitro reconstitution experiments were performed in Assay buffer (20 mM Tris, 100 mM NaCl, pH 7.5) in 50 µL total reaction volume and incubated at RT (20 – 22 °C), unless stated otherwise. An NADPH (10 mM) stock solution was freshly prepared and stored on ice until its addition to the reactions. Aliquots of enzymes and malonyl-CoA stocks were defrosted on ice as required whereas other components were stored at RT. Components were added to the reaction tube in a particular sequence: Assay Buffer > $MgCl_2$ > enzymes > malonyl-CoA > NADPH. Once all components were added, the reaction mixtures were loosely covered with foil to prevent product degradation from light. Negative controls were set up using either RerA-C216A (KS-inactivated mutant) instead of active RerA, or RerB-AxA (PPTase-inactivated mutant) instead of active RerB.

Monitoring of *in vitro* iPKS activity by UV-Vis spectroscopy

Reactions were set up as above in a 96-well clear flat bottom microplate. Once NADPH was added, the reaction was immediately monitored by Hidex Sense Microplate Reader by scanning in the range of 220 – 700 nm at 1 min interval for 60 min.

Use of photolabile probes in assays with recombinant iPKS enzymes

Photolabile chemical probes **5-6** (20 mM in DMSO) were irradiated with a home built light box equipped with a circular 22 W UVA lamp (365 nm) for 45 min to generate the active probes **7-8** ahead of their use in iPKS *in vitro* assays.

Each reaction was performed in Assay buffer (20 mM Tris, 100 mM NaCl, pH 7.5) containing RerA (20 µM), RerB or RePPT (20 µM), photolysed chemical probe **7** or **8** (400 or 2000 µM), malonyl-CoA (400 µM), NADPH (800 µM) and $MgCl_2$ (5 mM) in a total volume of 50 µL. Components were added to the assay tube in the following order: Assay Buffer > $MgCl_2$ > enzymes > photolysed chemical probe > malonyl-CoA > NADPH. Negative controls were set up using RerA-C216A (KS-inactivated mutant) instead of active RerA; RerB-AxA (PPTase-inactivated mutant) instead of active RerB; or addition of DMSO instead of chemical probe. The reactions were incubated at room temperature for 55 min then quenched and extracted twice by addition of EtOAc (100 µL), vortexed and centrifuged (21,000 *g*, 5 min, RT). The combined organic layers were evaporated to dryness by placing tubes in a desiccator connected to a high vacuum line. The residue was then redissolved in 150 µL of MeOH (HPLC grade) and stored at –20 °C until its analyses by LTQ Orbitrap Fusion instrument (usually within 48 h post reaction set up).

Purification and characterisation of orange enzyme-bound species from protein coexpression in *E. coli*

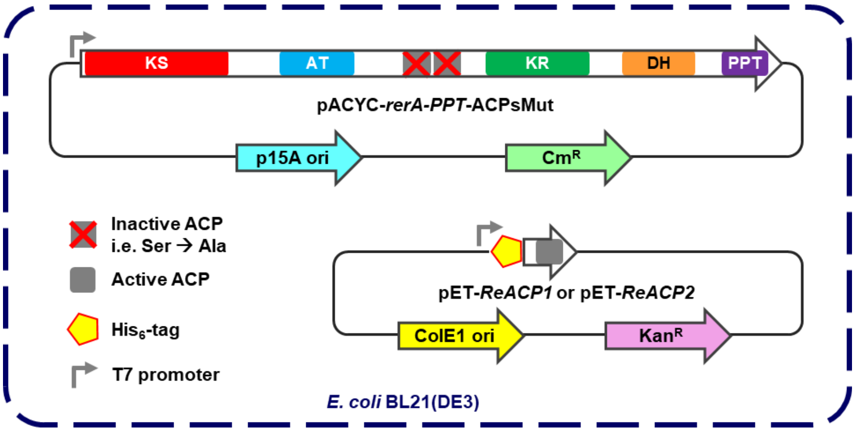

Figure S20 Illustration of protein *trans* coexpression system

For the coexpression experiment, two compatible plasmids were cotransformed into *E. coli* BL21(DE3), one encoding for an ACP-inactivated RerA-PPT for which both ACPs had been inactivated by SDM and the His_6-tag was not present (pACYC-rerA-PPT-ACPsMut plasmid); and the other plasmid encoding for active ACP domain bearing His_6-tag (p28T-ReACP1 or p28T-ReACP2). Orange cell lysate obtained from the coexpression of pACYC-rerA-PPT-ACPsMut (no His_6 tag) and p28T-ReACP1 or p28T-ReACP2 (with His_6 tag) was loaded onto a HisTrap HP 1 mL column and His_6-tagged recombinant ACP domains were eluted per the protocol described above. An aliquot of eluted His_6-tagged protein was taken directly for intact protein MS.

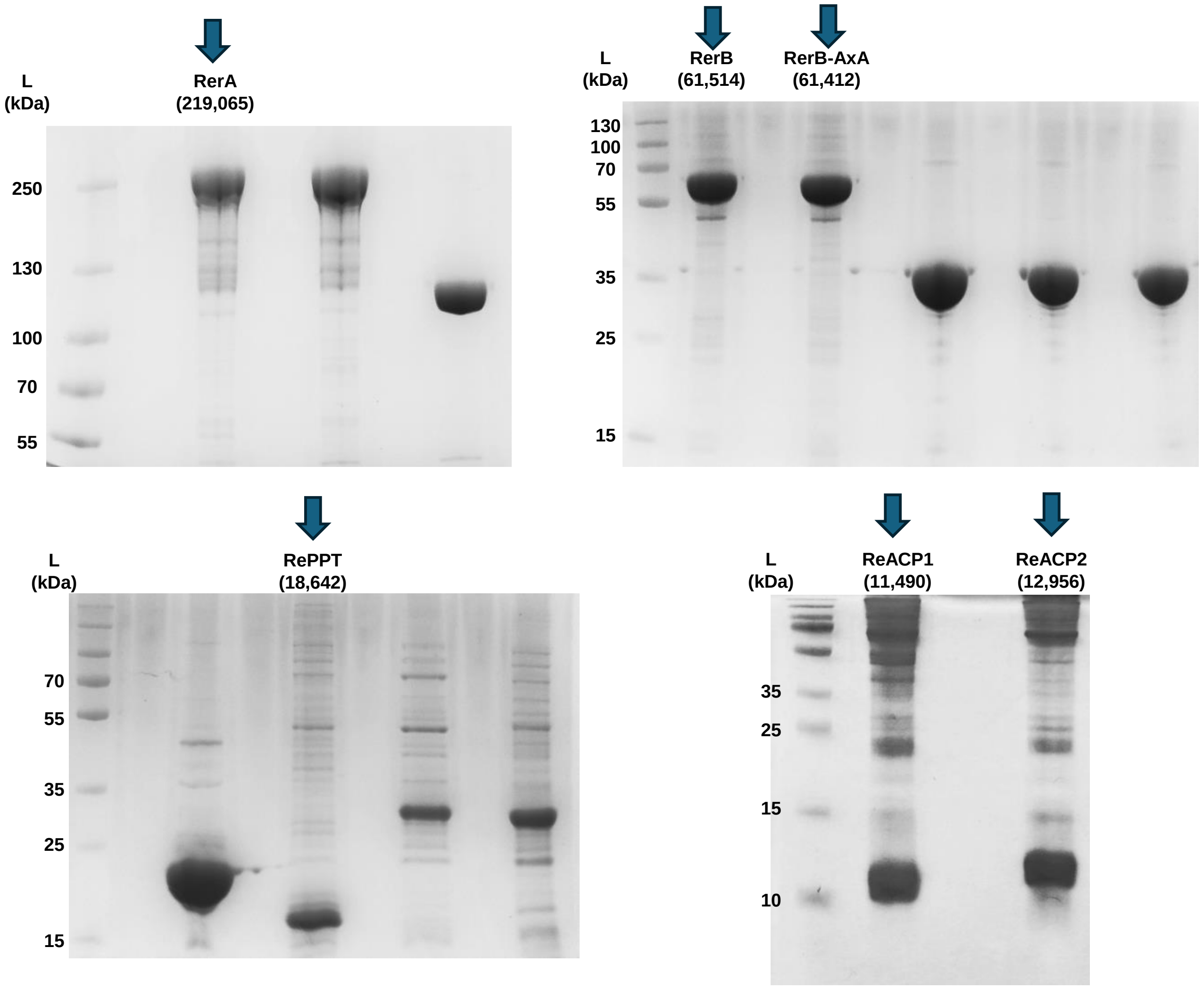

Figure S21 Uncropped original SDS-PAGE gel images for the recombinant proteins expressed and utilised in this study (highlighted by blue arrows).

LC-MS^n analyses

Analyses of recombinant proteins and coloured ACP domains

Purified recombinant proteins were analysed on Bruker MaXis II electrospray ionisation time-of-flight mass spectrometer (ESI-TOF-MS) using a Dionex 3000 RS UHPLC fitted with an ACE C4-300 RP column (100 x 2.1 mm, 5 µm). Flow rate for ACE C4-300 RP column was 0.2 mL/min and the elution profile is given below.

Time (min)	Buffer A% (H_2O + 0.1% formic acid)	Buffer B% (MeCN + 0.1% formic acid)
0	95	5
5	95	5
35	0	100
45	0	100

Analyses of small molecules

Samples containing small molecules (<2000 Da) were analysed on either Bruker Compact or Bruker MaXis Impact UHR-TOF mass spectrometer using a Dionex 3000 RS UHPLC fitted with a Zorbax Eclipse Plus C18 column (100 x 2.1 mm, 1.8 µm). Flow rate for Zorbax Eclipse Plus C18 column was 0.2 mL/min and the elution profile is detailed below.

Time (min)	Buffer A% (H_2O + 0.1% formic acid)	Buffer B% (MeCN + 0.1% formic acid)
0	80	20
5	80	20
27	0	100
45	0	100

Orbitrap Fusion analyses of small molecules

Reverse phase chromatography was used to separate the mixtures prior to MS analysis. Two columns were utilised: an Acclaim PepMap µ-precolumn cartridge 300 µm i.d. x 5 mm 5 µm 100 Å and an Acclaim PepMap RSLC C18 75 µm x 15 cm 2 µm 100 Å (Thermo Scientific). The columns were installed on an Ultimate 3000 RSLCnano system (Dionex). Samples were loaded onto the µ-precolumn equilibrated in 50% Buffer A after which compounds were eluted onto the analytical column following a 45 min gradient detailed below. Flow rate for Acclaim PepMap RSLC C18 column was 0.3 µL/min. Eluting cations were converted to gas phase ions by electrospray ionisation and analysed. Survey scans of precursors from 150 to 1500 m/z were performed at 60K resolution (at 200 m/z) with a 4 x 10^5 ion count target. Tandem MS was performed by isolation at 1.6 Th with the quadrupole, HCD fragmentation with normalized collision energy of 32, and rapid scan MS analysis in the ion trap. The MS^2 ion count target was set to 2 x 10^5 and the maximum injection time was 50 ms. A filter targeted inclusion mass list was used to select the precursor ions.

Time (min)	Buffer A% (H_2O + 0.1% formic acid)	Buffer B% (MeCN + 0.1% formic acid)
0	50	50
5	50	50
15	20	80
38	20	80
39	50	50
45	50	50

Protein sequences of *R. erythropolis* PR4-related constructs generated for this work

Underlined sequence represents His_6 tag and peptides encoded on the destination plasmid.

p28T-rerA (KS-AT-ACP1-ACP2-KR-DH)

MW: 219,065 Da

<u>MGSSHHHHHHSSGENLYFQGH</u>MTEDSTNPAIAIVGMSMWSPGAHDLQGFWENVLARRMQF
RKFPESRMSLDDYWSASPDDVDKTYADRGAFMDGFEFDWVGRRIPERTFKSTDLTHWLAL
ETALGALADAGYSRGSVPTGRSAAIVGNSLTGEESRMWSMRLRWPYVKRALAVAAEARQL
DSSVADALAETMEEVYKGPLVEPSEDTLAGMLSNTIAGRICNYLDFNGGGYVVDGA**C**ASG
MLAVATAAEKLASGAADFVLAGGVDVSLDPLELVGFARLGALTRGDMNVYDASRSGFIPG
EGCGFVALKRLEDARADGDYVYATIRGWGISTDGKGGITKPRAETQAEMIRRAYSGAGFA
ASEVAFVEGHGTGTPVGDPVELAGVQQAAQTDGPVEARSIGMTSLKSLIGHTKAASGILA
LIKATMAVNQRILPPLAGCTDPNPAFGTEAPALFPLVNGEIRDPSEKMRAGAQAMGFGGI
NCHVAIESADAPSSKLTPSQDVRTMMASYQDTEVFVLSADSAVDLATRARDVADLAVPLS
VAELLDFSAKLSRVISPTAPFRAAVVAGRPAQLAERMRQLAVICENSAPASGQVKVVSNE
ISISNSVRHDIGFLFPGQGSQQLEMARVLIERFEWARELATKADGWLEGVGAEAITPRIL
RNPVKSADAAELAKWKRDLAQTQFTQPAVALASLLWFEYLRRLGVTPSAVAGHSLGELTA
LYAAGAYDQKTLITLAAAKGAAMAVSGGGNGAMASLTCDRSTAEAIIAEAKGYATVANLN
TPTQSVVSGTKDAVDDVVAIAKTRGVSAQALAVSNAFHSEMMNEAERELKSTAPVEEQVD
SLTCPVYSCVEGERVQTPLALREFVTKQVVSPVDWVKTVSGISQEVDLLVEVGPGRVLTG
LTKAINGTDGVRCFPVASKSGRDEDFNVALAAMYVHGAQVRWNELFDGRFVREFVPADQK
VFIENLAEAKLTVTSAPEPLALGTSGGDPAAALADYLSRRGTFLVDVIRADVGSGATSPS
SPSVPSAPTSRAVTNGHESVAAKAPSPVSAPVVEEPAVAGAGSVESELIRILADTTGFPA
ESITTDLRLLDDLNLD<u>**S**</u>ISAAEAISKVAHQFEVVDLDPAELANATIGEAASLILAASPHP
GSAVPAASSAQSDVSRILLEVIAEDTGFPVESLDVDLHLLDDLNMD<u>**S**</u>IKAADAIATVATR
LGVQGDLDPAELVNVSLGELIEVLDRSAKEKSGQQQPAARALPAKAKGISADSFTWVRDY
TLEKVRSETALHGVRDLTDTTFVVIGDDAGLVGRLESELRGRGAQVDIRTFSDSTTANAH
HVVAVMPRVTDDSVTESGLRRGIEHMYKIVPAQQSGGARTTLAVVQFTDVELSNPAPVPS
VAAFTASVHHERPELDVRVIGFDGSTADAEVAATVVSELDSVAPYVFATYDAEMGRTALQ
PRVLASNDYTVRESGLGSSDVVVVTGGAKGIMAQCALALGRKTGAELVLIGSSQRQAGDE
IASTLADFTSAGLSAHYYRCNVTDAAAVAGVVAQIEVEVGSITGFVHGAGANVPRRFERV
DSAAAFKEVAPKVLGAANFVEALADRDLKLFVGFSSIIGFTGMPGNSWYAYGNELLDEMV
VRYAAGHPKTRTFSLAYSVWGETGMGARMGSVNHLAKMGVMPISTAAGVDHFLRLVDSGP
EASRIVVTSRLGGLDTWAPAAPALPAVSRYIDQVVTFEPQVELVTRTTLSTSADPFVLDH
VWKGSALLPTVFGLEAMSQAAAYVTGRVTLGRVRIDDIKLDRPIVVDTVEGTRVEIKASV
VEQDRDAAGTRVHVTIGTERTGYGRPHFSADFIFGLDESLPEFEKELPRPVLDIDPLDDL
YSWLLFQEGDFRRLEEISSLDSEHILFSAISRADRKQHLLGDPYFLDSLLQSGQIMVPRE
ICLPVNIARIDMYDGRFEARSFTAYAYDKVQTETHMQADVAVVKDGRVVMQLEGYRSQIL
SHDESRPTAEEIADPTARDAQIILDKLAQHSRALGVKSPRVVVAHTPGIHELTKSERHEQ
EKPIAEAAVNIHLDEDVVIK

C216 (active Cys on KS domain) is highlighted in cyan.
<u>S1076</u> (active Ser on ACP1 domain) is underlined in <u>red</u>.
<u>S1166</u> (active Ser on ACP2 domain) is underlined in <u>blue</u>.

pET28a-rerB (PPTase-Y-TE)

MW: 61,514 Da

MGSSHHHHHHSSGLVPRGSHMASMTGGQQMGRGSGDQMSNAFAIEWQETGKPVVVGDIPP
SVDVSLSHDGSKSLSSAGDWPQGC**D**I**E**PIRSRTVVQWKALLGSARIPLLDELIAGGESLD
RAGTRLWAAAEAIRKATGQMSVSLAMGAREDDAVTFRGGAEGSLVVLTFPALLTDNEERI
LAFVTDEADAAERVSDEADAEEPAQGPAPQFDSGPLSAKSLAQFDPMAYGSDVHVVARSG
YPAVSIRFPVGFRDACNVGGSVGFASFALWLGALRERGTGPISKQIVEDMATGRWGMVTN
NSEVFIDENLYTDDIVEGTIWITELTGPDRATTNLHVEWCRVDGETRTHIGWSAMQTTWV
EILSHGVVAARPMPQYFHDFIEPMTIPTGAKNIAVPTQPKDVDRGSLIRSAPMVPRNPYL
LESEVFSTTMQDGNVVGNIYFGNYYIWQSRV**R**DKFLARSQREAMAARGALGELRCVHTRV
EHLREVMPFDDVLVTMSLAALYERGIDLEFEYFKVNQDGSREKLAIARHRTVWTMPGAPG
ELEISPPSKLPQSLIDSVLEAIDGK

D48 and E50 (active binding residues on PPTase domain) are highlighted in pink.
R415 (residue on TE domain proposed to be involved in substrate binding) is highlighted in yellow.

p28T-rerAB (fused)

MW: 276,931 Da

GSGSG linker between RerA and RerB is highlighted in green.

MGSSHHHHHHSSGENLYFQGHMTEDSTNPAIAIVGMSMWSPGAHDLQGFWENVLARRMQF
RKFPESRMSLDDYWSASPDDVDKTYADRGAFMDGFEFDWVGRRIPERTFKSTDLTHWLAL
ETALGALADAGYSRGSVPTGRSAAIVGNSLTGEESRMWSMRLRWPYVKRALAVAAEARQL
DSSVADALAETMEEVYKGPLVEPSEDTLAGMLSNTIAGRICNYLDFNGGGYVVDGACASG
MLAVATAAEKLASGAADFVLAGGVDVSLDPLELVGFARLGALTRGDMNVYDASRSGFIPG
EGCGFVALKRLEDARADGDYVYATIRGWGISTDGKGGITKPRAETQAEMIRRAYSGAGFA
ASEVAFVEGHGTGTPVGDPVELAGVQQAAQTDGPVEARSIGMTSLKSLIGHTKAASGILA
LIKATMAVNQRILPPLAGCTDPNPAFGTEAPALFPLVNGEIRDPSEKMRAGAQAMGFGGI
NCHVAIESADAPSSKLTPSQDVRTMMASYQDTEVFVLSADSAVDLATRARDVADLAVPLS
VAELLDFSAKLSRVISPTAPFRAAVVAGRPAQLAERMRQLAVICENSAPASGQVKVVSNE
ISISNSVRHDIGFLFPGQGSQQLEMARVLIERFEWARELATKADGWLEGVGAEAITPRIL
RNPVKSADAAELAKWKRDLAQTQFTQPAVALASLLWFEYLRRLGVTPSAVAGHSLGELTA
LYAAGAYDQKTLITLAAAKGAAMAVSGGGNGAMASLTCDRSTAEAIIAEAKGYATVANLN
TPTQSVVSGTKDAVDDVVAIAKTRGVSAQALAVSNAFHSEMMNEAERELKSTAPVEEQVD
SLTCPVYSCVEGERVQTPLALREFVTKQVVSPVDWVKTVSGISQEVDLLVEVGPGRVLTG
LTKAINGTDGVRCFPVASKSGRDEDFNVALAAMYVHGAQVRWNELFDGRFVREFVPADQK
VFIENLAEAKLTVTSAPEPLALGTSGGDPAAALADYLSRRGTFLVDVIRADVGSGATSPS
SPSVPSAPTSRAVTNGHESVAAKAPSPVSAPVVEEPAVAGAGSVESELIRILADTTGFPA
ESITTDLRLLDDLNLDSISAAEAISKVAHQFEVVDLDPAELANATIGEAASLILAASPHP
GSAVPAASSAQSDVSRILLEVIAEDTGFPVESLDVDLHLLDDLNMDSIKAADAIATVATR
LGVQGDLDPAELVNVSLGELIEVLDRSAKEKSGQQQPAARALPAKAKGISADSFTWVRDY
TLEKVRSETALHGVRDLTDTTFVVIGDDAGLVGRLESELRGRGAQVDIRTFSDSTTANAH
HVVAVMPRVTDDSVTESGLRRGIEHMYKIVPAQQSGGARTTLAVVQFTDVELSNPAPVPS
VAAFTASVHHERPELDVRVIGFDGSTADAEVAATVVSELDSVAPYVFATYDAEMGRTALQ
PRVLASNDYTVRESGLGSSDVVVVTGGAKGIMAQCALALGRKTGAELVLIGSSQRQAGDE
IASTLADFTSAGLSAHYYRCNVTDAAAVAGVVAQIEVEVGSITGFVHGAGANVPRRFERV
DSAAAFKEVAPKVLGAANFVEALADRDLKLFVGFSSIIGFTGMPGNSWYAYGNELLDEMV
VRYAAGHPKTRTFSLAYSVWGETGMGARMGSVNHLAKMGVMPISTAAGVDHFLRLVDSGP
EASRIVVTSRLGGLDTWAPAAPALPAVSRYIDQVVTFEPQVELVTRTTLSTSADPFVLDH
VWKGSALLPTVFGLEAMSQAAAYVTGRVTLGRVRIDDIKLDRPIVVDTVEGTRVEIKASV
VEQDRDAAGTRVHVTIGTERTGYGRPHFSADFIFGLDESLPEFEKELPRPVLDIDPLDDL
YSWLLFQEGDFRRLEEISSLDSEHILFSAISRADRKQHLLGDPYFLDSLLQSGQIMVPRE
ICLPVNIARIDMYDGRFEARSFTAYAYDKVQTETHMQADVAVVKDGRVVMQLEGYRSQIL

SHDESRPTAEEIADPTARDAQIILDKLAQHSRALGVKSPRVVVAHTPGIHELTKSERHEQ
EKPIAEAAVNIHLDEDVVIK**GSGSG**SNAFAIEWQETGKPVVVGDIPPSVDVSLSHDGSKS
LSSAGDWPQGCDIEPIRSRTVVQWKALLGSARIPLLDELIAGGESLDRAGTRLWAAAEAI
RKATGQMSVSLAMGAREDDAVTFRGGAEGSLVVLTFPALLTDNEERILAFVTDEADAAER
VSDEADAEEPAQGPAPQFDSGPLSAKSLAQFDPMAYGSDVHVVARSGYPAVSIRFPVGFR
DACNVGGSVGFASFALWLGALRERGTGPISKQIVEDMATGRWGMVTNNSEVFIDENLYTD
DIVEGTIWITELTGPDRATTNLHVEWCRVDGETRTHIGWSAMQTTWVEILSHGVVAARPM
PQYFHDFIEPMTIPTGAKNIAVPTQPKDVDRGSLIRSAPMVPRNPYLLESEVFSTTMQDG
NVVGNIYFGNYYIWQSRVRDKFLARSQREAMAARGALGELRCVHTRVEHLREVMPFDDVL
VTMSLAALYERGIDLEFEYFKVNQDGSREKLATGAIARHRTVWTMPGAPGELEISPPSKL
PQSLIDSVLEAIDGK

p28T-RePPT (standalone PPTase domain from RerB)

Amino acid 1 to 154 of RerB; MW: 18,643 Da

<u>MGSSHHHHHHSSGENLYFQGH</u>MSNAFAIEWQETGKPVVVGDIPPSVDVSLSHDGSKSLSS
AGDWPQGCDIEPIRSRTVVQWKALLGSARIPLLDELIAGGESLDRAGTRLWAAAEAIRKA
TGQMSVSLAMGAREDDAVTFRGGAEGSLVVLTFPALLTDNEERILAFVTDEADAA

p28T-ReACP1 (standalone ACP1 domain from RerA)

Amino acid 1042 to 1129 of RerA; MW: 11,491 Da

<u>MGSSHHHHHHSSGENLYFQGHM</u>SVESELIRILADTTGFPAESITTDLRLLDDLNLDSISA
AEAISKVAHQFEVVDLDPAELANATIGEAASLILAASPHPGSAVPAASSA

p28T-ReACP2 (standalone ACP2 domain from RerA)

Amino acid 1131 to 1229 of RerA; MW: 12,956 Da

<u>MGSSHHHHHHSSGENLYFQGHM</u>SDVSRILLEVIAEDTGFPVESLDVDLHLLDDLNMDSIK
AADAIATVATRLGVQGDLDPAELVNVSLGELIEVLDRSAKEKSGQQQPAARALPAKAKGI
S

References

[1] G. Castro-Falcón, N. Millán-Aguiñaga, C. Roullier, P. R. Jensen, C. C. Hughes, *ACS Chem. Biol.*, 2018, **13**, 3097–3106.

[2] I. Wilkening, S. Gazzola, E. Riva, J. S. Parascandolo, L. Song, M. Tosin, *Chem. Commun.,* 2016, **52**, 10392–10395.

[3] S. L. Kilgour, R. Jenkins, M. Tosin, *Chem. Eur. J.,* 2019, **25**, 16511–16514.

[4] E. Riva, I. Wilkening, S. Gazzola, W. M. A. Li, L. Smith, P. F. Leadlay, M. Tosin, *Angew. Chem. Int. Ed.,* 2014, **53**, 11944–11949.

[5] K. Blin, S. Shaw, H. E. Augustijn, Z. L. Reitz, F. Biermann, M. Alanjary, A. Fetter, B. R. Terlouw, W. W. Metcalf, E. J. N. Helfrich, G. P. van Wezel, M. H. Medema, T. Weber, *Nucleic Acids Res.,* 2023, **51**, W46–W50.

[6] F. Madeira, M. Pearce, A. R. N. Tivey, P. Basutkar, J. Lee, O. Edbali, N. Madhusoodanan, A. Kolesnikov, R. Lopez, *Nucleic Acids Res.,* 2022, **50**, W276–W279.

[7] X. Robert, P. Gouet, *Nucleic Acids Res.,* 2014, **42**, W320–W324.

[8] C. L. M. Gilchrist, T. J. Booth, B. van Wersch, L. van Grieken, M. H. Medema, Y.-H. Chooi, *Bioinform. adv.,* 2021, **1**, 1–10.
